# *DDERMMAL*, a melanocytic long non-coding RNA, confers DNA damage tolerance to melanoma cells

**DOI:** 10.1016/j.isci.2026.116251

**Published:** 2026-06-05

**Authors:** Sara Adnane, Yvessa Verheyden, Alessandro Cuomo, Tiziana Bonaldi, Eleonora Leucci

**Affiliations:** 1 Laboratory for RNA Cancer Biology, Department of Oncology, KU Leuven, 3001 Leuven, Belgium; 2 IEO, European Institute of Oncology IRCCS, Via Adamello 16, 20139 Milan, Italy; 3 Department of Oncology and Hematology-Oncology, University of Milan, Milan 20122, Italy

**Keywords:** Cancer systems biology, Cell biology

## Abstract

Despite remarkable advances in targeted therapies and immunotherapies, a subset of patients fails to respond due to intrinsic resistance, and the majority of those who initially benefit eventually relapse. The emergence of resistant clones under therapeutic pressure has been attributed to both genetic and/or non-genetic resistance mechanisms. Understanding the mechanisms of drug tolerance that drive persistence and resistance, as well as identifying novel biomarkers and therapeutic vulnerabilities, is therefore critically important. Many long non-coding RNAs (lncRNAs) have been implicated in DNA damage; however, their role in melanoma, a malignancy characterized by high mutational rates, remains elusive. Here, we identify the lncRNA *DDERMMAL* as a modulator of DNA damage response (DDR) signaling. Our findings indicate that *DDERMMAL* enables melanoma cells to withstand genetic insults while sustaining proliferation, providing a mechanistic basis for lineage-specific persistence under therapeutic pressure and the emergence of *de novo* mutations driving genetic relapse.

## Highlights

• *DDERMMAL* is a novel melanocytic lincRNA regulated by SOX10 and MITF
• *DDERMMAL* interacts with the DNA damage-sensing and repair machinery
• *DDERMMAL* confers tolerance to PARP inhibition in melanocytic melanoma states

## Graphical abstract

**Figure:**
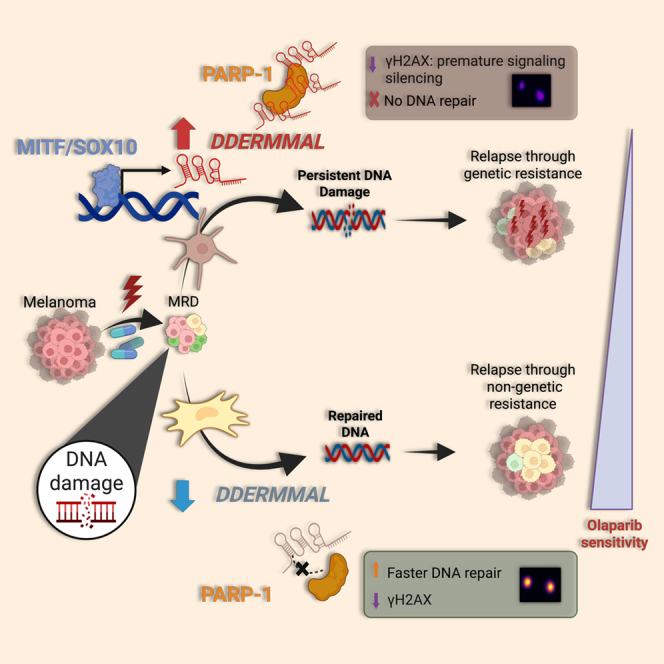

## Introduction

Over the past two decades, breakthroughs in targeted therapies and immunotherapies have transformed the management of advanced melanoma, leading to a substantial decrease in mortality. Today, BRAF and MEK inhibitors, along with combinatorial immune-based treatment using both anti-CTLA-4 (ipilimumab) and anti-PD-1 (nivolumab), represent the most effective targeted and immunotherapy strategies in the field, achieving 5-year survival rates of approximately 34% and 52%, respectively.^1^^,^^2^ Despite these encouraging outcomes, most advanced melanomas remain incurable, as a large proportion of patients either fail to respond or ultimately relapse. Notably, fewer than 20% of patients treated with BRAF/MEK inhibitors experience long-term responses exceeding five years^1^, and approximately 20–30% of patients relapse within 1 to 2 years of starting anti-PD-1-based treatment.^3^ The challenge of achieving durable remission is largely driven by the emergence of acquired resistance mechanisms, as well as the frequent failure to initiate a therapeutic response due to intrinsic resistance, particularly in the context of immunotherapy.^4^^,^^5^^,^^6^^,^^7^

While much of the current understanding of melanoma drug tolerance focuses on phenotype switching into dedifferentiated, slow-cycling drug-tolerant cell states at minimal residual disease (MRD), such as neural crest stem cell (NCSC)-like and mesenchymal-like phenotypes,^8^^,^^9^^,^^10^ recent studies based on single-cell RNA sequencing of melanoma patient-derived xenograft models following MAPK-targeted therapy have identified a pigment-producing, drug-tolerant cluster within MRD lesions, characterized by high activity of MITF, the master regulator of melanocyte differentiation and pigmentation, and increased expression of pigmentation/differentiation markers such as MLANA, DCT and TRPM1, representing an alternate path toward drug tolerance and resistance.^8^ Consistently, ∼50% of melanoma tumors relapsing through acquired resistance retain MITF activity comparable to, or even exceeding, pre-treatment levels,^11^^,^^12^ and patients with pigmented metastatic melanomas display significantly reduced overall and disease-free survival compared to those with non-pigmented tumors.^13^^,^^14^ Furthermore, enhanced MITF activity has been linked to intrinsic resistance.^12^^,^^15^^,^^16^^,^^17^ These observations parallel reports from metastasis studies showing that melanoma cells dedifferentiate and exhibit reduced proliferative activity during invasion and metastasis, but regain a differentiated, melanocytic/pigmented proliferative phenotype once they reach secondary sites.^18^^,^^19^ These melanocytic states, often overlooked in studies of melanoma resistance and generally regarded as drug-sensitive, may persist during therapy and/or reemerge following transcriptional reprogramming, thereby potentially contributing to disease progression and poor prognosis.^8^^,^^20^^,^^21^

Interestingly, recent work by Yang et al. demonstrated that melanoma cells capable of rapidly escaping BRAF inhibition and proliferating under drug pressure accumulate DNA damage yet outcompete other subpopulations over time.^22^ The persistence of such DNA-damaged, proliferating cells is expected to increase the likelihood of mutagenesis and the acquisition of *de novo* mutations that can ultimately drive drug resistance.^22^ This risk is heightened in melanoma, a malignancy inherently characterized by high mutational rates due to ultraviolet (UV) exposure,^23^^,^^24^ oncogene-induced replication stress^25^, and alterations in the DNA damage response (DDR) signaling pathways,^26^^,^^27^^,^^28^ all factors that are further exacerbated under therapeutic pressure.^28^^,^^29^

LncRNAs are transcripts longer than 200 nucleotides, increasingly recognized as pivotal players in tumorigenesis and as key mediators of adaptive responses in cancer progression and during therapeutic resistance.^30^^,^^31^ They exert their functions through mechanisms such as sponging or scaffolding of biomolecules, enabled by their inherent multivalency, structural flexibility, and frequent enrichment in repetitive sequences.^32^ LncRNAs have a clearly recognized role in DDRs.^33^^,^^34^ However, their contribution to DNA damage tolerance and subsequent therapy resistance, particularly in the context of melanoma, a cancer affected by high mutational rates, remains poorly understood. Here, we identify *DDERMMAL*, a novel melanocytic lineage-enriched lncRNA, as a modulator of DDR signaling. Our findings suggest that *DDERMMAL* contributes to the ability of melanocytic melanoma cells to withstand genotoxic stress while maintaining proliferative capacity, thereby providing a new mechanistic framework for understanding lineage-specific persistence under therapeutic pressure.

## Results

### *DDERMMAL* is highly expressed in melanoma, and its increased expression is associated with poor survival

**Figure 1:**
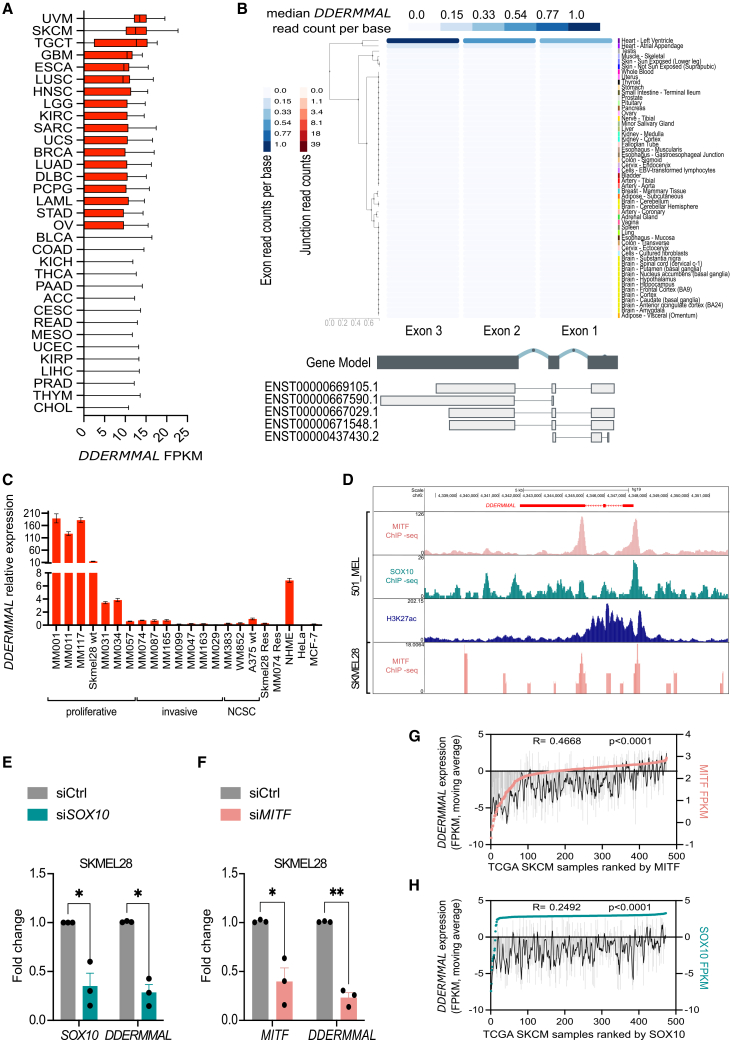
*DDERMMAL* is a melanocytic lineage-associated lincRNA regulated by MITF and SOX10 and highly expressed in melanoma (A) *DDERMMAL* expression in the PanCancer Atlas expressed in Fragments per Kilobase Per Million (FPKM). (B) *DDERMMAL* expression in a panel of adult healthy tissues from the GTEx database. (C) Relative expression of *DDERMMAL* in a panel of melanoma lines with different mutations and representative of different drug-tolerant states, as well as in NHME, HeLa, and MCF7 cells, as calculated by RT-qPCR. Error bars represent standard deviations (SDs) of qPCR replicates (*n* = 2). (D) UCSC genome browser snapshot of *DDERMMAL* locus with ChIP-seq tracks for MITF, SOX10, and H3K27ac in 501 Mel cells.^35^^,^^37^ MITF ChIP-seq data from SKMEL28 melanoma cells^36^ are also shown. (E) Relative expression of *SOX10* and *DDERMMAL* in SKMEL28 upon knockdown of *SOX10*. Data are shown as mean ± SEM from independent biological replicates (*n* = 3). Statistics were calculated by multiple paired *t* test. (F) Relative expression of *MITF* and *DDERMMAL* in SKMEL28 upon knockdown of *MITF*. Data are shown as mean ± SEM from independent biological replicates (*n* = 3). Statistics were calculated by multiple paired *t* test. (G) Correlation analysis of *DDERMMAL* and *MITF* expression in 473 melanoma samples (TCGA SKCM). (H) Correlation analysis of *DDERMMAL* and *SOX10* expression in 473 melanoma samples (TCGA SKCM).

We set out to characterize the lncRNA AL159166.1 (Ensembl ID: ENSG00000231811), also known as LOC101927888, which we renamed *DDERMMAL* for DNA damage enabling regulator, melanocytic melanoma-associated lncRNA. We selected this specific candidate as *in silico* analysis of *DDERMMAL* expression in the TCGA Pan-Cancer dataset revealed its nearly exclusive association with melanoma, with the two highest expressing cancers being uveal melanoma (UVM) and skin cutaneous melanoma (SKCM) (Figure 1A), while in healthy tissues, *DDERMMAL* expression was restricted to the heart (Figure 1B). Expression profiling across a panel of melanoma cell lines and normal melanocytes (NHME) revealed high levels of *DDERMMAL* in melanocytic cell lines and NHME, and low or absent expression in mesenchymal-like and NCSC-like lines (Figure 1C). Examination of *DDERMMAL* locus using publicly available ChIP-seq data revealed binding sites for the melanocytic lineage-specific transcription factors SOX10 and MITF, upstream of its transcription start site (TSS) in 501 Mel cells^35^ (Figure 1D). MITF binding at this upstream region was also detected in ChIP-seq data from SKMEL28 melanoma cells^36^ (Figure 1D). These binding regions overlap with H3K27ac marks in 501 Mel cells,^37^ consistent with active promoters/enhancers (Figure 1D). In line with its downregulation in dedifferentiated melanoma subpopulations, knockdown of *SOX10* and *MITF* using siRNA pools significantly reduced *DDERMMAL* expression (Figure 1E-F), indicating that this lncRNA is transcriptionally regulated by lineage-specific factors. Consistently, the analysis of *DDERMMAL*, *MITF,* and *SOX10* expression across melanoma samples (TCGA SKCM) revealed that *DDERMMAL* positively correlates with both TFs (Figure 1G-H). At the molecular level, *DDERMMAL* is a polyadenylated lincRNA (Figure S1A), comprising 5 isoforms (Figure 1B) and localizing equally to the cytoplasm and nucleus (Figures S1B–S1C).

**Figure 2:**
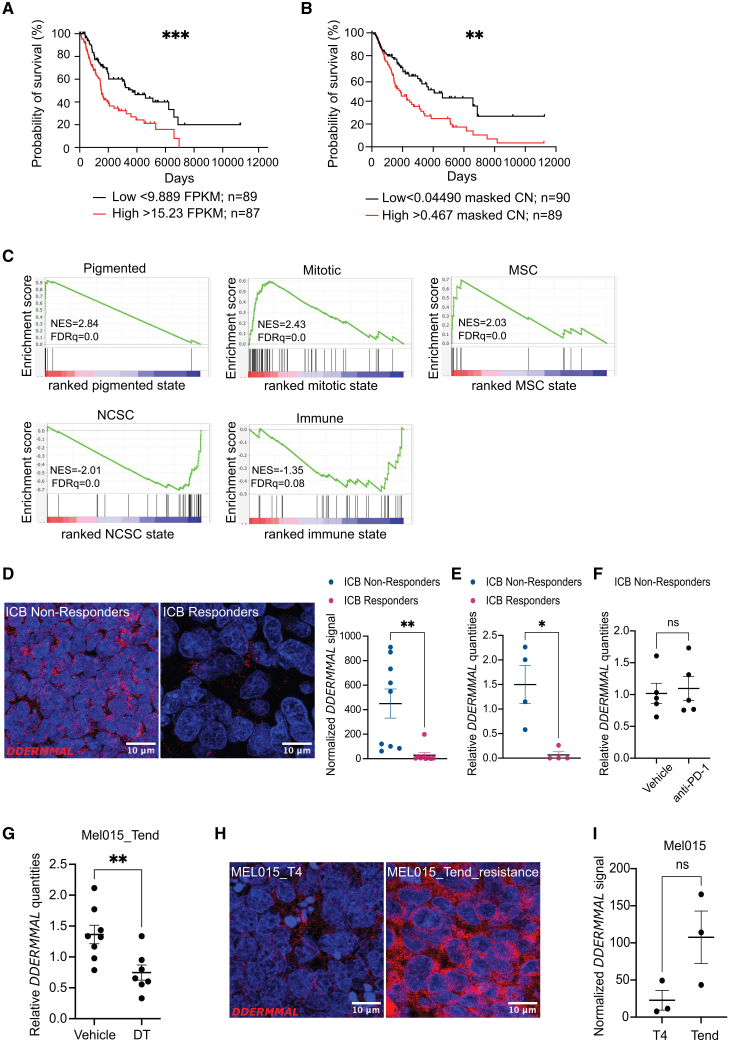
Increased *DDERMMAL* expression is associated with poor survival in melanoma (A) Probability of survival of patients with melanoma stratified based on *DDERMMAL* expression. Log rank (Mantel-Cox) test was performed to calculate statistical significance. (B) Probability of survival of patients with metastatic melanoma stratified based on *DDERMMAL* copy number. Log rank (Mantel-Cox) test was performed to calculate statistical significance. (C) Gene set enrichment analysis (GSEA) of melanoma transcriptional states in patients stratified into “*DDERMMAL*-high” and “*DDERMMAL*-low” groups based on median *DDERMMAL* expression. NCSC: neural crest stem cell-like; MSC: mesenchymal; NES: normalized enrichment score; FDR: false discovery rate. (D) FISH for *DDERMMAL (*red) in patient-derived xenograft (PDX) tumor models responding or progressing under immune checkpoint blockade (ICB responders and non-responders), derived from the same patient tumor and propagated in independent mice. Nuclei are counterstained with DAPI (blue). Scale bar = 10 μm. The graph on the right shows the quantification of *DDERMMAL* signal in the corresponding tumors. Three independent biological replicates (*n* = 3) were analyzed, with three images per replicate; each dot represents one image. Values are expressed as integrated density normalized to total tissue area (mean ± SEM). (E) RT-qPCR analysis of relative *DDERMMAL* expression in vehicle-treated tumors from ICB responders and non-responders at relapse (*n* = 4 mice derived from the same patient tumor). Data are shown as mean ± SEM. Statistical significance was determined using an unpaired *t* test. (F) RT-qPCR of relative *DDERMMAL* expression in vehicle- or anti-PD-1-treated PDX tumors from ICB non-responders at relapse. Data are shown as mean ± SEM from independent biological replicates (*n* = 5 mice derived from the same patient tumor). Statistical significance was calculated by unpaired *t* test. (G) RT-qPCR of relative *DDERMMAL* expression in vehicle- or targeted therapy (dabrafenib + trametinib)–treated PDX tumors at relapse. Data are shown as mean ± SEM from independent biological replicates (vehicle, *n* = 8; DT, *n* = 7). Statistical significance was calculated by the unpaired *t* test. (H) FISH for *DDERMMAL* (red) in Mel015 PDX tumors treated with targeted therapy (dabrafenib + trametinib), shown 4 days after treatment initiation (T4) and at relapse (Tend). Nuclei are counterstained with DAPI (blue). Scale bar = 10 μm. I. Quantification of *DDERMMAL* signal in tumors shown in H. Three independent biological replicates (*n* = 3) were analyzed, with two images per biological replicate, and each dot representing the replicate mean. Values are expressed as integrated density normalized to total tissue area (mean ± SEM). Statistical significance was calculated using an unpaired *t* test.

Kaplan-Meier survival analysis of metastatic melanoma (GDC TCGA) revealed that high *DDERMMAL* expression and copy number were significantly associated with poor patient survival (Figure 2A–B). Stratification of tumors into “*DDERMMAL*-high” and “*DDERMMAL*-low” groups based on median expression, followed by gene set enrichment analysis (GSEA) of melanoma transcriptional states defined by Rambow et al. (2018), showed that *DDERMMAL* expression significantly and inversely correlates with the NCSC and the immune state (Figure 2C).

As melanoma standard of care includes immune checkpoint blockade (ICB), to further assess the relevance of our findings, we examined by fluorescent *in situ* hybridization (FISH) *DDERMMAL* expression in humanized melanoma PDX models. *DDERMMAL* was significantly upregulated in an actively growing, ICB-refractory PDX model, compared to an ICB responder model (Figure 2D-E). Furthermore, qPCR for *DDERMMAL* in vehicle-treated and anti-PD-1-treated mice indicated that its expression is not affected by ICB (Figure 2F).

In targeted therapy (dabrafenib and trametinib)-treated PDX models, qPCR analysis indicated that *DDERMMAL* expression was significantly lower at relapse when compared to vehicle controls (Figure 2G), consistent with the downregulation of melanocytic programs by MAPK inhibition. However, within DT-exposed tumors, a clear trend toward higher *DDERMMAL* levels was observed at T_relapse_ (drug-resistant cells) compared to T_4_ (drug-tolerant cells after 4 days exposure to treatment) (Figure 2H-I), although this difference did not reach statistical significance (*p* = 0.08), suggesting a re-establishment of melanocytic programs that may enable proliferative expansion at relapse. Taken together, these findings position *DDERMMAL* as a marker of melanocytic, proliferative, immune-cold persistent tumors.

### *DDERMMAL* is critical for melanocytic cell viability, growth, and invasion

**Figure 3:**
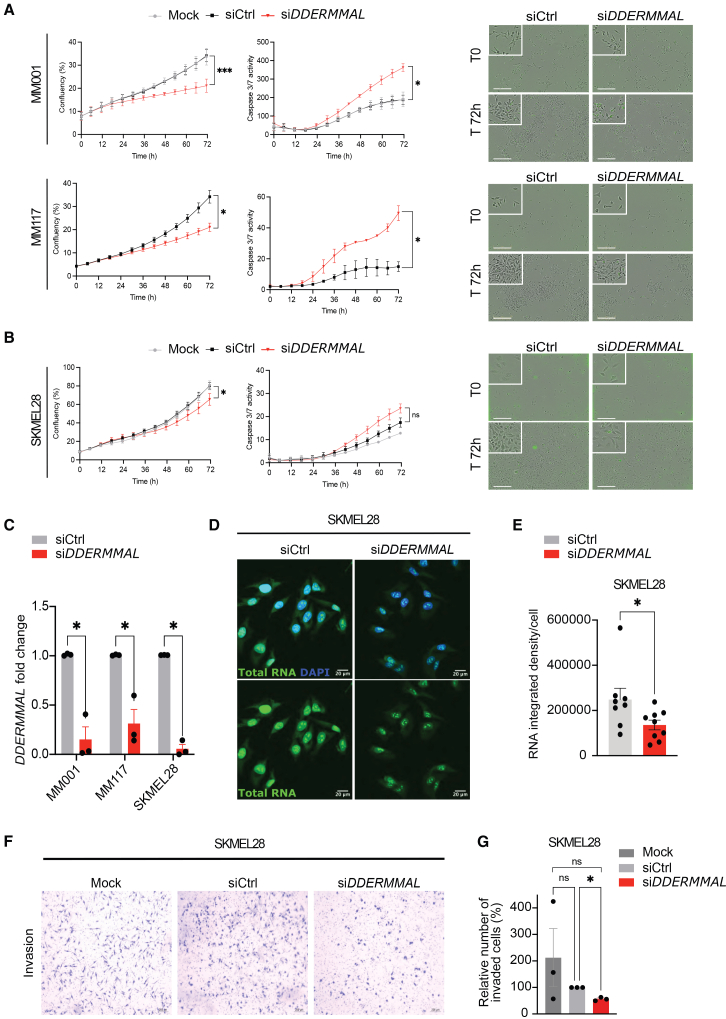
*DDERMMAL* is critical for melanocytic cell viability, growth, and invasion (A) Cell confluency (left) and caspase 3/7 activation (middle) measured by live-cell imaging in MM001 and MM117 cells upon *DDERMMAL* knockdown. Brightfield images (right) are representative of the cells right after transfection (T0) and 72 h post-transfection (T72h). Scale bar = 300 μm. Data are shown as mean ± SEM from independent biological replicates (*n* = 3). Statistical significance was assessed by two-way ANOVA with mixed-effects analysis. (B) Cell confluency (left) and caspase 3/7 activation (middle) measured by live-cell imaging in SKMEL28 cells upon *DDERMMAL* knockdown. Brightfield images (right) are representative of the cells right after transfection (T0) and 72 h post-transfection (T72h). Scale bar = 300 μm. Data are shown as mean ± SEM from independent biological replicates (*n* = 3). Statistical significance was assessed by two-way ANOVA with mixed-effects analysis. (C) RT-qPCR showing *DDERMMAL* knockdown efficiency in melanoma cell lines, expressed as fold change relative to control. Data are shown as mean ± SEM from independent biological replicates (*n* = 3). Statistical significance was calculated using multiple paired t-tests. (D) Total RNA staining (green) upon *DDERMMAL* knockdown in SKMEL28. The upper panel shows the merged image of RNA staining and DAPI (blue), while the lower panel shows RNA staining alone. Representative images from 3 independent biological replicates are shown. Scale bar = 20 μm. (E) Quantification of the images in F expressed as mean integrated density per cell (mean ± SEM). For the quantification, 9 images from 3 independent biological replicates (*n* = 3) were used, with each dot representing one image. Statistics were calculated by the unpaired *t* test. (F) Matrigel invasion assay of SKMEL28 cells upon *DDERMMAL* knockdown. Representative images from 3 independent biological replicates are shown. Scale bar = 200 μm. (G) Quantification of the Matrigel invasion assay shown in D. Three independent biological replicates (*n* = 3) were analyzed, with three images per biological replicate, and each dot represents the replicate average. Values are expressed as mean ± SEM. Statistical significance was assessed by one-way ANOVA.

Using a siRNA pool targeting all isoforms of *DDERMMAL* (Figure S1E), we assessed the effect of its depletion on melanoma cell viability across lines representing different melanocytic programs. *DDERMMAL* knockdown in the high-MITF lines, MM001 and MM117, the two highest expressors, induced rapid cell death (Figures 3A–3C). By contrast, in SKMEL28, an intermediate *DDERMMAL* expressor with high MITF activity, depletion caused a significant reduction in confluency without increased apoptotic activity (Figures 3B, 3C; S1F-G), indicative of cell-cycle arrest. Consistent with their impaired proliferative capacity, a noteworthy decrease in total RNA signal was observed in SKMEL28 (Figure 3D-E). As total RNA staining predominantly reflects ribosomal RNA, whose nucleolar assembly occurs during late telophase and throughout interphase and is disassembled upon entry into mitosis,^38^ this reduction further supports the presence of a cell-cycle arrest phenotype. Conversely, the mesenchymal-like line MM165 and the NCSC-like line A375, both representative of dedifferentiated states and low *DDERMMAL* expression, were unaffected by its knockdown (Figure S1H). These results suggest that *DDERMMAL* is selectively essential in melanocytic and/or pigmented, mitotic, melanoma cells, consistent with the clinical association between high *DDERMMAL* expression and poor patient survival. Furthermore, SKMEL28 cells exhibited a significant reduction in invasive capacity upon *DDERMMAL* depletion, underscoring its role in promoting aggressive features of melanoma (Figure 3F-G).

These results prompted us to test whether *DDERMMAL* depletion induces a loss of pigmentation and a switch toward a dedifferentiated state. Nonetheless, pigmentation markers (*MLANA, DCT,* and *TYRP1*) and NCSC markers (*AQP1, NGFR,* and *L1CAM*) remained unchanged upon knockdown, indicating that *DDERMMAL* is neither a pigmentation effector nor a driver of dedifferentiation trajectories, but rather a melanocytic lineage-essential lncRNA (Figure S1I).

### *DDERMMAL* interacts with the DNA damage/repair machinery

**Figure 4:**
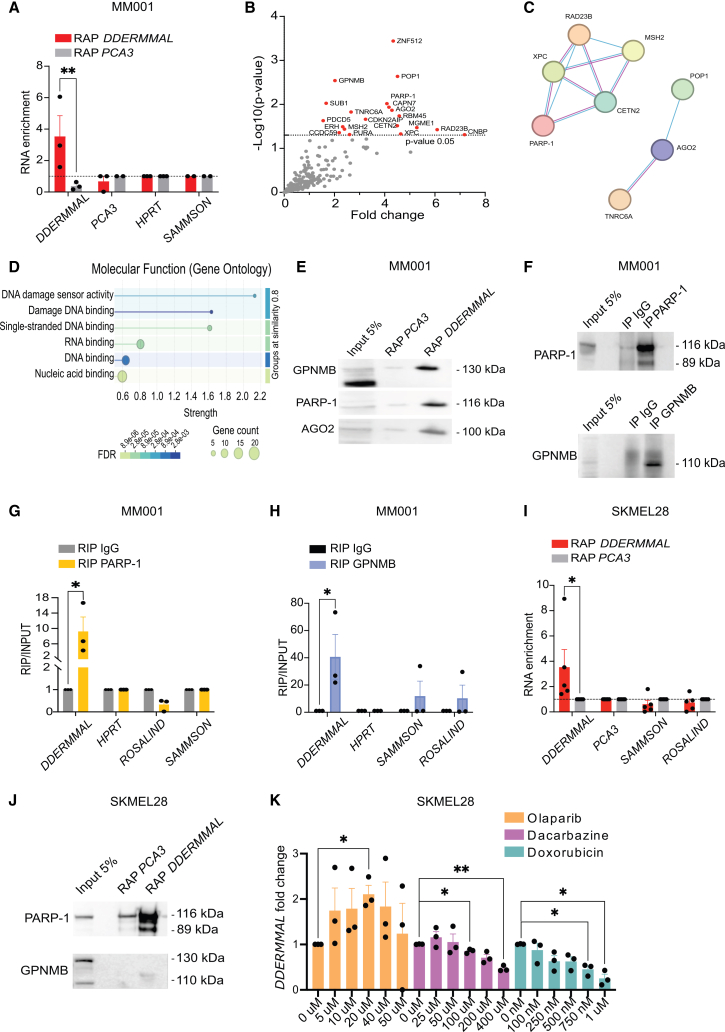
*DDERMMAL* interacts with the DNA damage/repair machinery (A) *DDERMMAL* enrichment over input in control RNA Affinity Purification (*PCA3* RAP) and in *DDERMMAL-specific* RAP (*DDERMMAL* RAP) as calculated by RT-qPCR. *HPRT* and *SAMMSON* were used as negative controls. Data are shown as mean ± SEM from independent biological replicates (*n* = 3). Statistics were calculated by ANOVA. (B) Protein enriched in *DDERMMAL*’s pull-down. Red dots indicate proteins significantly enriched (fold change>1.5 *p* < 0.05). (C) Protein-protein interaction (PPI) network of the selected gene set generated using the STRING database.^39^ Each node represents a protein encoded by the input genes, and edges indicate experimentally determined or predicted functional associations. Line thickness reflects the confidence score of the interaction, as provided by STRING. (D) Gene Ontology (molecular function) enrichment analysis of the selected gene set was performed using the STRING database. Enriched GO terms were grouped by semantic similarity (threshold = 0.8) to reduce redundancy. The x axis indicates the enrichment strength, calculated on a log10 scale as a ratio between the observed and expected gene counts. Circle size represents the number of genes associated with each term, and color intensity corresponds to the false discovery rate (FDR <0.05). (E) Western blot validation of GPNMB, PARP-1, and AGO2 as *DDERMMAL* partners in MM001. (F) Control of PARP-1 and GPNMB IP by western blot validation. Normal IgGs have been used as a control. Images are representative of 3 independent biological replicates. (G) *DDERMMAL* enrichment over input in control (RIP IgG) and PARP-1-specific RNA Immune Precipitation (RIP PARP-1) as calculated by RT-qPCR. *HPRT, ROSALIND,* and *SAMMSON* have been used as negative controls. Data are shown as mean ± SEM from independent biological replicates (*n* = 3). Statistics were calculated by ANOVA. (H) *DDERMMAL* enrichment over input in control (RIP IgG) and GPNMB-specific RNA immune precipitation (RIP GPNMB) as calculated by RT-qPCR. *HPRT, ROSALIND,* and *SAMMSON* were used as negative controls. Data are shown as mean ± SEM from independent biological replicates (*n* = 3). Statistics were calculated by ANOVA. (I) *DDERMMAL* enrichment over input in SKMEL28 upon UV crosslinking and RNA Affinity Purification with control (*PCA3* RAP) and *DDERMMAL-*specific probes (*DDERMMAL* RAP) as calculated by RT-qPCR. *ROSALIND* and *SAMMSON* were used as negative controls. Data are shown as mean ± SEM from independent biological replicates (*n* = 4). Statistics were calculated by multiple paired *t* test. (J) Western blot validation of PARP-1 and GPNMB as *DDERMMAL* partners in SKMEL28. (K) Change in *DDERMMAL* expression indicated as fold change upon the treatment of SKMEL28 with several DNA-damaging agents for 24 h. Data are shown as mean ± SEM from independent biological replicates (*n* = 3). Statistics were calculated by ANOVA.

To dissect *DDERMMAL*’s mechanism of action, we searched for its protein interactors. Toward this, we designed biotinylated probes against *DDERMMAL* and performed RNA antisense purification (RAP), followed by mass spectrometry analysis of interacting proteins in MM001 (Figure 4A-B), given the essential role of *DDERMMAL* in the survival of this high-expressing, melanocytic cell line (Figures 1C; 3A; Table S1). As a control, we used a probe set against *PCA3*, a lincRNA not expressed in melanoma. MS-based analysis led to the identification of 219 proteins, among which 20 were significantly enriched in the *DDERMMAL* pulldown fraction (fold change>1.5 *p* < 0.05) (Figure 4B) compared to the control lncRNA. STRING network analysis^39^ of the proteins significantly enriched in the *DDERMMAL* pulldown (Figure 4C), followed by Gene Ontology functional enrichment analysis (Figure 4D), revealed functionally associated clusters, with the predominant cluster mainly involved in DDRs. Supporting this functional link, *DDERMMAL* expression was sensitive to osmotic changes, which have been shown to increase R Loops formation and DNA damage.^40^ Specifically, *DDERMMAL* was found to be downregulated by hypoosmotic stress induced by culture medium dilution and upregulated by hyperosmotic stress induced by the exposure to 100 mM sucrose treatment (Figure S1D).

Together, these findings suggest that *DDERMMAL* is dynamically regulated by genotoxic stress and may act at the interface between DNA damage induction and its recognition by PARP-1.

To further investigate *DDERMMAL*’s molecular function, three of the protein targets identified by RAP-Mass Spectrometry proteomics analysis were validated by western blot (Figure 4E). While PARP-1, a key DNA damage sensor, and GPNMB, a melanocytic cell marker regulated by MITF implicated in pigmentation,^41^ immune suppression,^42^ and metastasis,^43^ were validated also by RNA immunoprecipitation (Figures 4F–4H) followed by western blot, AGO2 was not further investigated. Of note, all 5 *DDERMMAL*’s isoforms were enriched in PARP-1 immune precipitates (Figure S2A), indicating that the smallest isoform of 329 nt may be the “minimal functional *DDERMMAL.*” The interaction with PARP-1 was also validated in SKMEL28 by RAP in UV crosslinking conditions, followed by western blot (Figure 4I-J). As for GPNMB, the interaction in this cell line could be confirmed only in 1 out of 4 biological replicates (Figure 4J), indicating that the interaction is cell-line specific.

*DDERMMAL* expression was downregulated in a dose-dependent manner upon the treatment of SKMEL28 with two different DNA-damaging agents, dacarbazine and doxorubicin, and upregulated upon the inhibition of PARP-1 DNA repair functions with olaparib (Figure 4K).

Importantly, *DDERMMAL* knockdown did not alter PARP-1 cleavage in any of the cell lines tested, including MM001, indicating that the loss of viability in this cell line is not mediated by apoptotic PARP-1 processing (Figure S2B-C). Total PARP-1 protein levels were markedly decreased in MM001, most likely as a consequence of cell death, whereas in other cell lines, levels appeared steady or only slightly reduced, as shown by western blot and immunofluorescence (Figures S2B–S2L). RNA-FISH combined with immunofluorescence revealed a moderate spatial overlap between *DDERMMAL* and PARP-1, which was only slightly reduced upon *DDERMMAL* knockdown, while the localization of PARP-1 remained unaltered (Figures S2D–S2L). Collectively, these findings suggest that *DDERMMAL* does not affect PARP-1 cleavage or subcellular distribution but may instead influence its functional engagement in DNA repair processes.

### *DDERMMAL* modulates the balance between DNA damage signaling and repair and promotes tolerance to PARP-1 inhibition

**Figure 5:**
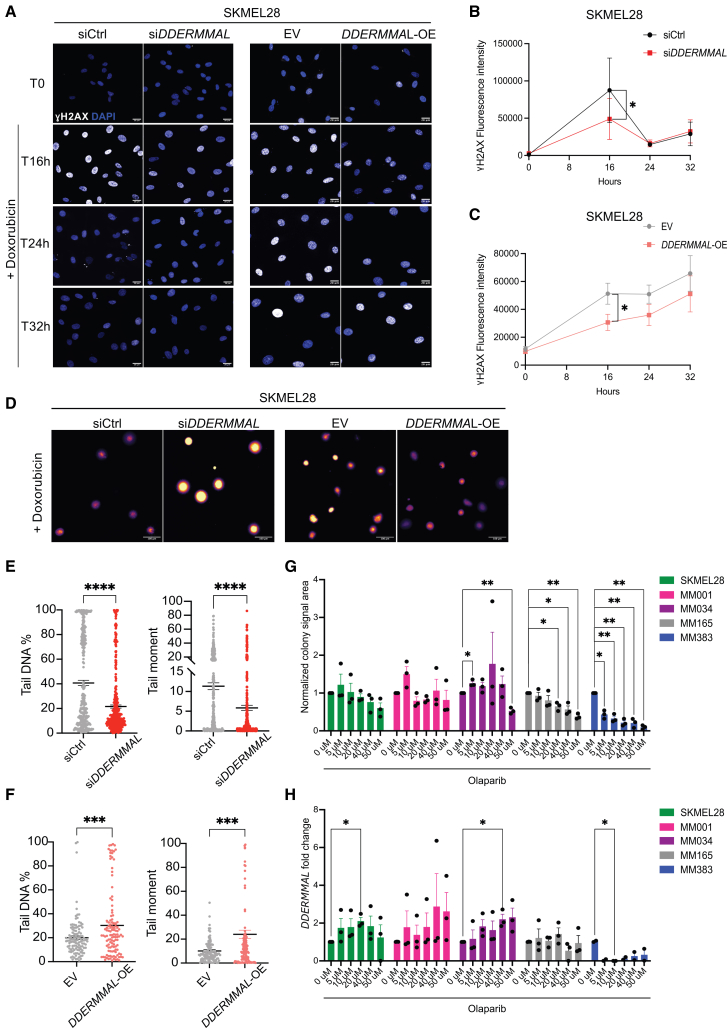
*DDERMMAL* modulates the balance between DNA damage signaling and repair and promotes tolerance to PARP-1 inhibition (A) Immunofluorescence of γH2AX (white) in SKMEL28 cells upon *DDERMMAL* knockdown (si*DDERMMAL*) or stable overexpression (*DDERMMAL*-OE), alongside their respective controls (siCtrl and empty vector, EV), following a 250 nM doxorubicin pulse. Timepoints indicate hours after doxorubicin administration. Nuclei were counterstained with DAPI (blue). Representative images from 3 independent biological replicates are shown. Scale bar = 20 μm. (B) Quantification of γH2AX signal in SKMEL28 upon *DDERMMAL* knockdown during time-course doxorubicin treatment, expressed as mean integrated density per cell. Data are shown as mean ± SEM from independent biological replicates (*n* = 4). Statistical significance was assessed using two-way ANOVA. (C) Quantification of γH2AX signal in SKMEL28 stably overexpressing *DDERMMAL* during time-course doxorubicin treatment, expressed as mean integrated density per cell. Data are shown as mean ± SEM from independent biological replicates (*n* = 3). Statistical significance was assessed using two-way ANOVA. (D) Comet assay of SKMEL28 cells upon *DDERMMAL* knockdown (si*DDERMMAL*) or stable overexpression (*DDERMMAL*-OE), with their respective controls (siCtrl and empty vector, EV), following 16 h exposure to 250 nM doxorubicin. Representative images from 3 independent biological replicates are shown. Scale bar = 100 μm. (E and F) Quantification of tail DNA% and tail moment from comets in D. Each dot represents a single comet. Data are shown as mean ± SEM from independent biological replicates (*n* = 3). Statistical significance was assessed by an unpaired *t* test. (G) Quantification of the viability assay of different melanoma cell lines upon exposure to increasing concentrations of Olaparib for 24 h (shown in Figure S4), expressed as area covered by the crystal violet staining normalized on the untreated control, per cell line. Data are shown as mean ± SEM from independent biological replicates (*n* = 3 per cell line). Statistics were calculated by ANOVA. (H) *DDERMMAL* expression as fold change relative to the non-treated sample in the experiment in A, as calculated by RT-qPCR. Data are shown as mean ± SEM from independent biological replicates (*n* = 3 per cell line). Statistics were calculated by ANOVA.

To investigate the role of *DDERMMAL* in DDRs, we knocked it down in SKMEL28 cells and quantified DNA damage by γH2AX staining and comet assay at different timepoints following a doxorubicin pulse. *DDERMMAL* knockdown significantly reduced γH2AX signal 16 h post-treatment (Figure 5A-B), a timepoint at which comet assays also revealed a significant reduction in tail DNA% and tail moments (Figure 5D-E), consistent with enhanced repair. Conversely, overexpression of the minimal isoform reduced γH2AX accumulation (Figures 5A–5C) while significantly increasing comet tail DNA% and moments (Figures 5D–5F), indicative of greater DNA damage accumulation and pointing to an uncoupling of DNA damage signaling and repair. Of note, overexpression of the minimal isoform alone did not affect cell viability (Figure S3) but was sufficient to alter the coordination between damage signaling and repair, highlighting this minimal isoform as a core functional unit of *DDERMMAL*.

To test whether this regulatory role extends to DNA repair inhibition, we exposed multiple lines to increasing concentrations of Olaparib, a PARP-1 inhibitor. The melanocytic lines MM001, SKMEL28, and MM034 exhibited little loss of viability except at the highest dose (50 μM), while consistently showing the upregulation of *DDERMMAL* (Figures 5G-H; S4). In contrast, mesenchymal-like (MM165) and NCSC-like (MM383) lines, which express very low levels of *DDERMMAL*, displayed marked, dose-dependent sensitivity to PARP inhibition, accompanied by unchanged or further reduced *DDERMMAL* expression (Figures 5G-H; S4). These results indicate that the induction of *DDERMMAL* is specific to the melanocytic state and associated with relative tolerance to PARP inhibition, indicating that melanocytic melanoma cells may exploit *DDERMMAL* to withstand DNA damage accumulation to persist under therapeutic stress.

## Discussion

Here, we identified *DDERMMAL*, a melanocytic lineage-associated lincRNA regulated by MITF and SOX10, which interacts with the DNA damage/repair machinery. *DDERMMAL* is essential for the survival and proliferation of melanocytic, drug-naive states, and this dependency strengthens with increasing melanocytic identity. MITF-high melanocytic cells are characterized by active pigmentation programs, including melanogenesis, which generate reactive oxygen species (ROS) and contribute to elevated oxidative stress and DNA damage.^44^ In addition, these melanocytic states are more proliferative, and rapid cycling is intrinsically linked to replication stress, further suggesting these states are predisposed to higher baseline genotoxic stress compared to mesenchymal-like or NCSC-like states.

Functionally, *DDERMMAL* enables melanocytic states to tolerate genotoxic stress while maintaining growth by uncoupling DNA damage signaling from repair through its interaction with PARP-1. Recent research has provided evidence of direct functional interactions between PARP-1 and lncRNAs. For instance, the lncRNA *BGL3* is recruited by PARP-1 to sites of DNA double-strand breaks where it facilitates the retention of the BRCA1-BARD1 complex and thereby supports the homologous recombination (HR) repair.^45^ Additionally, the lncRNA *SPARCLE*, which is induced upon genotoxic stress, interacts with PARP-1, prompting its cleavage and subsequently triggering cell death.^46^ Here we report that *DDERMMAL* also interacts with PARP-1, although it does not affect its processing. We hypothesize that *DDERMMAL* may rather rewire its interactome to modulate DNA repair, although the precise mechanism of this engagement remains to be elucidated.

Notably, our results indicate that PARP-1 is not restricted to the nucleus. Previous studies have shown that PARP-1 translocates to the cytoplasm in response to DNA damage or viral infection, where it suppresses type I interferon responses.^47^ In melanoma, reduced type I interferon activity has been shown to antagonize ICB,^48^ a feature consistent with the immune-cold tumor phenotypes associated with high *DDERMMAL* expression.

Prior research has identified a pigmented subpopulation among MRD persisters under targeted therapy. While our work does not track persistence, we propose *DDERMMAL*-mediated tuning of DDRs as a novel mechanistic rationale for how melanocytic melanomas could withstand therapeutic/genotoxic pressure, and continue proliferating despite DNA lesions, thereby increasing the likelihood of accumulating *de novo* mutations responsible for relapse. Clinically, high-*DDERMMAL* metastatic melanomas are associated with poor prognosis and resistance, suggesting *DDERMMAL*-mediated damage tolerance may contribute to their adverse outcomes. MITF-high melanomas frequently display substantial genomic instability, reflected in elevated single-nucleotide and copy-number variant burdens, a phenotype linked to MITF’s role as a modulator of DDRs through its interaction with the MRN complex, in addition to its transcriptional control of cell cycle and repair genes.^49^ Consistent with this, earlier findings demonstrated that MITF supports melanoma cell survival under genotoxic stress by maintaining DNA replication and an effective damage response sufficient to prevent senescence.^50^

The selective vulnerability of melanoma cells with active melanocytic programs to *DDERMMAL* inhibition, which compromises their survival, highlights *DDERMMAL* as a potential Achille’s heel in pigmented/melanocytic melanomas, worthy of further exploration *in vivo*. At the same time, its low expression in dedifferentiated melanomas, together with the observed sensitivity of these states to the PARP-1 inhibitor Olaparib, suggests that *DDERMMAL* status could potentially inform state-specific therapeutic approaches: Dedifferentiated tumors may be more likely to respond to PARP inhibitors, whose therapeutic value in melanoma is currently under investigation either as monotherapies or in combination with immunotherapy (as listed on ClinicalTrials.gov), whereas melanocytic tumors could rely on *DDERMMAL*-mediated DNA damage tolerance to persist under genotoxic stress.

In this context, the induction of *DDERMMAL* upon olaparib treatment in drug-naïve cells that resist cell death supports a model in which *DDERMMAL* forms part of an adaptive DDR response, promoting DNA damage tolerance away from high-fidelity HR toward more permissive, error-prone end-joining repair pathways or tolerance mechanisms, thereby enabling cells to sustain proliferation under stress. Furthermore, the similarities observed in γH2AX immunofluorescence upon exposure to doxorubicin in both depletion and overexpression contexts suggest that its mechanism of action lies outside canonical HR/NHEJ repair execution and instead points toward a role in RNA-dependent DDR condensates emerging as key regulators of repair factor dynamics. Notably, RNA species generated at sites of DNA damage, including damage-induced long non-coding RNAs (dilncRNAs), which may be processed to short transcripts termed DNA damage response RNAs (DDRNAs) and functionally interact with them, have been shown to stimulate molecular crowding of damage response proteins such as 53BP1 in the shape of foci.^33^^,^^51^^,^^52^ Rather than acting as a static scaffold that recruits specific repair complexes, *DDERMMAL* may contribute to the formation and/or modulation of dynamic RNA-dependent DDR condensates, in which multivalent RNA-protein interactions regulate the local concentration, availability, and timing of repair factors downstream of PARP-1 activation. This possibility is further supported by the enrichment of *DDERMMAL* in repetitive sequences and by RAP-mass spectrometry results identifying, in addition to PARP-1, several DDR-associated interactors, including MSH2, XPC, RAD23B, and CETN2. Further supporting this model, recent studies have demonstrated that PARP-1 can co-condensate with DNA lesions to stabilize broken DNA ends and facilitate the recruitment and assembly of DNA repair effectors.^53^^,^^54^

Together, our findings identify *DDERMMAL* as a novel melanocytic lineage-associated lncRNA that plays a role in the DDR and proliferative resilience under genotoxic stress, while being linked to melanoma state-dependent vulnerabilities, providing a strong rationale for future mechanistic and translational investigation.

### Limitations of the study

This study provides multiple independent lines of evidence supporting a role for *DDERMMAL* as a melanocytic lineage-associated lncRNA involved in DDRs and melanoma state-dependent vulnerabilities. However, further work will be required to define its precise mechanistic mode of action, including how it modulates PARP-1–associated processes and DDR dynamics. In addition, the validation of its functional relevance *in vivo*, particularly in the context of tumor progression and therapeutic response, will be important to fully establish its role in melanoma biology.

## Resource availability

### Lead contact

Further information and requests for resources and reagents should be directed to and will be fulfilled by the lead contact, Eleonora Leucci (E-mail: eleonora.leucci@kuleuven.be).

### Materials availability

This study did not generate new unique reagents.

### Data and code availability

• Proteomics data have been deposited to the ProteomeXchange Consortium via the PRIDE: PXD060369.
• No custom code was used in this study. All analyses were performed using standard, publicly available software tools.
• Data generated in this paper are available from the lead contact upon reasonable request.

## Acknowledgments

This project was funded by C1 Interne Fondsen KU Leuven/C1 Internal Funds KU Leuven (C16/19/006). Yvessa Verheyden was an FWO – Flanders Research Organization PhD fellowship (1SC5122N). We thank Chiara Ghirardi for the technical support of the proteomics experiment and Prof. Ludo Van Den Bosch for kindly reviewing the manuscript.

## Author contributions

S.A.: conceptualization, experimental work, data analysis, visualization, methodology, and writing original draft. Y.V.: data analysis. T.B. and A.C.: data acquisition and analysis. E.L.: conceptualization, supervision, funding acquisition, and writing original draft.

## Declaration of interests

The authors declare no competing interest.

## STAR★Methods

### Key resources table

**Table:** 

REAGENT or RESOURCE	SOURCE	IDENTIFIER
**Antibodies**		

Rabbit anti-phospho-Histone H2A.X (Ser139)	Cell Signaling Technology	Cat# 2577; RRID:AB_2118010
Rabbit anti-PARP-1	Abcam	Cat# ab227244; RRID: AB_3676687
Mouse anti-GPNMB (clone D-9)	Santa Cruz Biotechnology	Cat# sc-271415; RRID: AB_10610660
Mouse anti-AGO2 (clone 11A9)	Gift from Gunter Meister	N/A
Rabbit anti-β-Actin (clone 13E5)	Cell Signaling Technology	Cat# 4970S; RRID: AB_2223172
HRP-linked anti-mouse IgG secondary antibody	Sigma-Aldrich	Cat# NA931; RRID: AB_772210
HRP-linked anti-rabbit IgG secondary antibody	Sigma-Aldrich	Cat# NA934; RRID: AB_772206
Alexa Fluor 647 anti-rabbit secondary antibody	Thermo Fisher Scientific	Cat# A32733; RRID: AB_2633282
Normal rabbit IgG	Millipore	Cat# 12-370; RRID: AB_145841

**Bacterial and virus strains**		

*Escherichia coli* NEB® 10-beta competent cells	New England Biolabs	Cat# C3019
Lentiviral particles encoding *DDERMMAL* (pLenti PGK Puro DEST)	This paper	N/A
Lentiviral particles (empty vector control, pLenti PGK Puro DEST)	This paper	N/A

**Biological samples**		

FFPE melanoma PDX tissue sections	KU Leuven, TRACE collection	https://gbiomed.kuleuven.be/english/research/50488876/54502087/Trace

**Chemicals, peptides, and recombinant proteins**		

Opti-MEM™ I Reduced Serum Medium	Thermo Fisher Scientific	Cat# 31985062
Lipofectamine™ 2000 Transfection Reagent	Invitrogen	Cat# 11668019
Puromycin	Invivogen	Cat# ant-pr-1
Dacarbazine	Selleckchem	Cat# S1221
Doxorubicin hydrochloride	Sigma-Aldrich	Cat# 44583
Olaparib (AZD2281)	Selleckchem	Cat# S1060
SYTO ® RNASelect™ Green Fluorescent Cell Stain	Thermo Fisher Scientific	Cat# S32703
Halt™ Protease and Phosphatase Inhibitor Cocktail	Thermo Fisher Scientific	Cat# 78442
SUPERase·In™ RNase Inhibitor	Thermo Fisher Scientific	Cat# AM2696
Pierce™ Streptavidin Magnetic Beads	Thermo Fisher Scientific	Cat# 88817
Dynabeads™ Protein A for Immunoprecipitation	Thermo Fisher Scientific	Cat# 10002D
Dynabeads™ Protein G for Immunoprecipitation	Thermo Fisher Scientific	Cat# 10004D
TRIzol™	Thermo Fisher Scientific	Cat# 15596018
Proteinase K	Sigma-Aldrich	Cat# P4850
Crystal Violet	Sigma-Aldrich	Cat# C0775
Dextran sulfate sodium salt	Sigma-Aldrich	Cat# 42867
Formamide	Sigma-Aldrich	Cat# F9037
Saline sodium citrate (20X)	Thermo Fisher Scientific	Cat# J60839.K2
ProLong™ Glass Antifade Mountant	Thermo Fisher Scientific	Cat# P36982

**Critical commercial assays**		

MycoAlert® Mycoplasma Detection Kit	Lonza	Cat# LT07-318
BioCoat® Matrigel® Invasion Chamber (8.0 μm PET membrane, 24-well)	Corning	Cat# 354480
High-Capacity cDNA Reverse Transcription Kit	Thermo Fisher Scientific	Cat# 4368813
CellEvent™ Caspase-3/7 Green Detection Reagent	Thermo Fisher Scientific	Cat# C10723
OxiSelect Comet Assay Kit	Cell Biolabs	Cat# STA-351
Nuclei EZ Prep Kit	Sigma-Aldrich	Cat# NUC101
GoTaq® G2 DNA Polymerase	Promega	Cat# M7848
PowerUp™ SYBR™ Green Master Mix for qPCR	Thermo Fisher Scientific	Cat# A25778
Gibson Assembly® Master Mix	New England Biolabs	Cat# E2611S
CalPhos™ Mammalian Transfection Kit	Takara	Cat# 631312

**Deposited data**		

Single-cell RNA-seq dataset of melanoma transcriptional states	Rambow et al.^8^	GSE116237
MITF and SOX10 ChIP-seq data from 501_Mel cells	Laurette, P. et al.^35^	GSE61967
MITF ChIP-seq data from SKMEL28	Kenny, C. et al.^36^	GSE190610
H3K27ac ChIP-seq data from 501_Mel cells	Fontanals-Cirera, B. et al.^37^	GSE94488
Proteomics data from *DDERMMAL*-RAP experiments	This paper	PRIDE: 1-20251031-100333-95870105

**Experimental models: Cell lines**		

Human: SKMEL28 melanoma cell line	ATCC	N/A
Human: A375 melanoma cell line	ATCC	CRL-1619
Human: MM383 and WM852 melanoma cell lines	Gift from Göran Jönsson	N/A
Human: patient-derived low-passage MM cell lines	Gift from G.-E. Ghanem	N/A
Human: HEK293 cell line	ATCC	CRL-1573
Normal Human Melanocytes (NHME)	Gift from M. Van Gele	N/A

**Oligonucleotides**		

ON-TARGETplus Human MITF siRNA – SMARTpool	Horizon Discovery	Cat# L-008674-00-0005
ON-TARGETplus Human SOX10 siRNA - SMARTpool	Horizon Discovery	Cat# L-017192-00-0005
Lincode Human LOC101927888 siRNA – SMARTpool	Horizon Discovery	Cat# R-191663-00-0005
ON-TARGETplus Non-targeting Control siPOOL	Horizon discovery	Cat# D-001810-10-05
Stellaris FISH probes targeting *DDERMMAL*	Biosearch Technologies	Sequences in Table S1
RAP probes targeting *DDERMMAL* and *PCA3*	Biosearch Technologies/IDT	Sequences in Table S1
qPCR primers	IDT	Sequences in Table S2

**Recombinant DNA**		

pLenti PGK Puro DEST (w529-2) -*SAMMSON* expression construct	GenScript; generated in Leucci et al.^55^	N/A
pLenti PGK Puro DEST empty vector (w529-2) (*SAMMSON* insert removed)	This paper	N/A
pLenti PGK Puro DEST (w529-2) -*DDERMMAL* expression construct	This paper	N/A

**Software and algorithms**		

GraphPad Prism version	GraphPad Software	v10.2.3; https://www.graphpad.com
Fiji	NIH	https://fiji.sc/
Big-FISH	Imbert et al.^56^	https://github.com/fish-quant/big-fish
OpenComet (ImageJ plugin)	GitHub	https://github.com/bgyori/opencomet
IncuCyte ZOOM software	Essen BioScience	N/A
MaxQuant	Max Planck Institute	v1.6.2.3
QuantStudio Design and Analysis Software	Thermo Fisher Scientific	v2.6.0
GSEA	Broad Institute	v4.3.3
STRING	STRING database	v12.0; https://string-db.org
UCSC Genome Browser	UCSC	https://genome.ucsc.edu/
UCSC Xena Browser	UCSC Xena	https://xena.ucsc.edu

### Experimental model and study participant details

#### Patient-derived xenograft (PDX) models

The cutaneous melanoma PDX models used for the assessment of *DDERMMAL* expression are part of the TRACE collection. Establishment and biobanking of these models were conducted in accordance with the Declaration of Helsinki, GDPR regulations, and institutional, national, and European guidelines for animal care and use. All procedures were approved by the KU Leuven Animal Ethics Committee (P164/2019) and the UZ Leuven/KU Leuven Research Ethics Committee (S63799).

#### Cell lines

Human melanoma cell lines SKMEL28 and A375 were obtained from ATCC. MM383 and WM852 melanoma cell lines were kindly provided by Göran Jönsson, patient-derived low-passage melanoma (MM) cell lines (MM034, MM011, MM001, MM074, MM087, MM057, MM163, MM029, MM099, MM165, MM031, MM162, MM047 and MM052) by G.-E. Ghanem, and normal human melanocytes (NHMEs) by M. Van Gele. All cell lines used were human-derived and routinely tested negative for mycoplasma using the MycoAlert Mycoplasma Detection kit (Lonza). They were cultured at 37°C and 5% CO2 in either RPMI 1640 (Gibco BRL Invitrogen) or F-10 (Gibco BRL Invitrogen) supplemented with 10% FBS (Gibco BRL Invitrogen) and 1% penicillin/streptomycin (Sigma-Aldrich). NHMEs were grown in MGM-4 melanocyte growth medium (Lonza). Cell lines were derived from patients of the following genders: Female (F) – MM034, MM011, MM001, MM074, MM087, A375, MM057, MM163, MCF-7, and MM029; Male (M) – SKMEL28, MM099, MM165, MM031, MM162, MM047, MM052, MM029, MM383, and WM852.

### Method details

#### Cell transfection and DNA damage treatment

For *DDERMMAL* knockdown experiments, cells were transfected with Lipofectamine 2000 (Invitrogen) in Opti-MEM reduced serum medium (Gibco) according to manufacturer instructions, either with 25 nM Non-targeting control siPOOL (Horizon Discovery) or with *DDERMMAL* targeting siRNA (Lincode Human LOC101927888 siRNA – SMARTpool, Horizon Discovery) 24h after seeding. For *MITF* and *SOX10* knockdown, the same procedure was followed with 50 nM Non-targeting siPOOL (Horizon Discovery) or with *MITF/SOX10* targeting siRNA (SMARTpool, Horizon Discovery).

For DNA damage induction or DNA repair inhibition assays, cells were treated with Dacarbazine (Cat# S1221, Selleckchem), Doxorubicin hydrochloride (Cat# 44583, Sigma-Aldrich), or Olaparib (Cat# S1060, Selleckchem) at the indicated concentrations, 24 h post-seeding.

#### Colony assays

To assess colony formation upon *DDERMMAL* knockdown or Olaparib treatment, cells were seeded in 6-well plates to reach approximately 50% confluency by the following day, at which point they were either transfected as previously described or treated with Olaparib (AZD2281, Selleckchem) at the indicated concentrations and incubated for an additional 72 h. Cells were then washed with 1× PBS (Sigma-Aldrich), fixed, and stained for 20 min using a solution of 1% crystal violet (Sigma-Aldrich) and 35% methanol (Thermo Fisher Scientific) in distilled water. Quantification of area coverage under each condition was performed using ImageJ.

#### Invasion assay

The invasive capacity of melanoma cells following *DDERMMAL* knockdown was assessed using a 24-h Matrigel invasion assay. SKMEL28 cells were transfected with either 25 nM non-targeting siPOOL (Horizon Discovery), 25 nM *DDERMMAL*-targeting siRNA SMARTpool (Horizon Discovery), or treated with Lipofectamine 2000 (Invitrogen) alone (mock control) to check for potential effects of the transfection reagent. 48 h post-transfection, cells were harvested and counted. For each condition, 25,000 cells were seeded into the upper compartment of a Corning BioCoat Matrigel Invasion Chamber (8.0 μm PET membrane, 24-well format, catalog no. 354480) in RPMI 1640 medium containing 1% penicillin/streptomycin. The remaining cells were used for RNA extraction to verify knockdown efficiency. The lower wells were filled with RPMI 1640 supplemented with 10% FBS and 1% penicillin/streptomycin, serving as a chemoattractant. After 24 h of incubation at 37°C and 5% CO_2_, non-invading cells on the upper surface of the membrane were gently removed using a wet cotton swab. Invaded cells were fixed, stained with crystal violet, and excess stain was removed according to the manufacturer’s recommendations. Imaging was performed using a Leica DFC295 microscope (5×/0.12 objective), with three fields of view acquired per insert. Quantification was performed using ImageJ, and invasion levels were normalized to the non-targeting control.

#### RNA extraction and RT-qPCR

RNA was extracted by collecting and homogenizing samples in 500 μL to 1 mL of TRIzol reagent (Thermo Fisher Scientific), followed by the addition of chloroform (Sigma-Aldrich) at 20% of the total volume. After a brief vortex, the mixture was centrifuged at 17,000 × g for 15 min at 4°C. The upper aqueous layer was transferred to a fresh tube, and RNA was precipitated using 0.5 mL of isopropanol (Fisher Scientific). Samples were mixed thoroughly and incubated at room temperature for 40 min, then centrifuged again at 17,000 × g for 30 min at 4°C. The resulting RNA pellet was washed twice with 70% ethanol, allowed to air-dry briefly, and dissolved in DEPC-treated, RNase-free water (Thermo Fisher Scientific).

To eliminate contaminating DNA, RNA was treated with 0.5 to 1 μL of DNase I (Invitrogen) in 10× DNase buffer and incubated for 20 min at 37°C, then placed on ice. An equal volume of phenol:chloroform (5:1; Sigma-Aldrich) was added, and samples were centrifuged at 17,000 × g for 15 min at 4°C. The aqueous phase was then collected and mixed with 1/6 volume of 5 M NaCl and 2.5 volumes of 100% ethanol for precipitation. Samples were stored at −80°C overnight and centrifuged the following day at 17,000 × g for 20 min at 4°C. The pellet was rinsed with 70% ethanol, centrifuged again, and finally resuspended in RNase-free water.

cDNA synthesis was carried out using the High-Capacity complementary DNA Reverse Transcription Kit (Thermo Fisher Scientific) according to the manufacturer’s instructions, using a Veriti 96-well thermal cycler (Thermo Fisher Scientific). RNA expression was quantified by perfoming quantitative PCR (qPCR) using PowerUp SYBR Green Master Mix (Thermo Fisher Scientific) on a QuantStudio 5 Real-Time PCR System (Thermo Fisher Scientific). Gene expression levels were quantified using the ΔΔCt method, with *GAPDH*, *TBP* and *UBC* serving as reference genes for normalization. Primer sequences used for qPCR are listed in Table S2. To assess polyadenylation levels of *DDERMMAL*, the same procedure of reverse transcription was executed using either random hexamers (Thermo Fisher Scientific) or an Oligo(dT)12–18 Primer (Thermo Fisher Scientific) following manufacturer’s instructions.

#### IncuCyte assays

For live cell imaging, cells were plated in 96 well-plates (TPP) and monitored by the IncuCyte ZOOM system (Essen BioScience) taking 4 images per well every 2 h. Cell death was assessed by adding the CellEvent Caspase-3/7 Green Detection Reagent (1:5000, Thermo Fisher Scientific) in the culture media. Cell confluency and caspase activity were measured and analyzed using the IncuCyte ZOOM software.

#### Fluorescent *In situ* hybridization

*DDERMMAL* RNA-FISH was performed using a 24 Stellaris FISH probeset (Biosearch Technologies) labeled with TAMRA C-9 dye and designed with the Stellaris probe designer software (Table S1). Cells were cultured on either round glass coverslips (VWR) or in PhenoPlate 96-well plates (PerkinElmer), fixed in 3.7% formaldehyde for 10 min at room temperature, and permeabilized in 70% ethanol overnight at 4°C. The following day, cells were washed twice with wash buffer (2× saline sodium citrate, 10% formamide) and were hybridized with a final probe concentration of 125 nM in hybridization buffer (2× saline sodium citrate, 10% formamide, and 10% dextran) overnight at 37°C. Subsequently, cells were washed with wash buffer for 30 min at 37°C then stained with DAPI (1:1000 in wash buffer, Sigma Aldrich). Finally, samples were mounted using Invitrogen ProLong Glass Antifade mounting media (Thermo Fisher Scientific). The same protocol was applied to FFPE sections, after an initial deparaffinization step at 65°C for 1 h, succeeded by two washes in xylene and rehydration in successive concentrations of 100%, 90%, 70%, and 40% ethanol and followed by an antigen retrieval step in a 10 μM sodium citrate buffer at pH 6, at 90°C for 30 min. Zeiss Celldiscoverer 7 Airyscan microscope was used for imaging *DDERMMAL*-FISH. FISH images were quantified using either the Big-FISH quant package^56^ or Fiji.

#### Immunofluorescence

For γH2AX immunofluorescence, cells were plated in μ-Slide 8 Well chambers (Ibidi). At the indicated timepoints, cells were washed with 1× PBS, fixed with 4% paraformaldehyde (PFA) for 20 min at room temperature (RT) under a chemical hood, and washed twice with PBS. Cells were stored in PBS at 4°C until all timepoints were collected and then stained as follows. Cells were permeabilized with 1% BSA (Sigma-Aldrich) and 0.2% Triton X-100 (Sigma-Aldrich) in PBS on ice for 10 min, followed by incubation at RT for 30 min in blocking buffer (10% goat serum in permeabilization buffer). Cells were then incubated with the primary antibody Phospho-Histone H2A.X (Ser139) (2577, Cell Signaling Technology), diluted 1:1000 in blocking buffer, for 1 h at RT. After incubation, cells were washed three times with permeabilization buffer (5 min each), then incubated in the dark for 45 min at RT with α-rabbit Alexa Fluor 647 secondary antibody (Thermo Fisher Scientific; 1:1000 in blocking buffer). Cells were washed three additional times and incubated with DAPI (1:1000, Sigma-Aldrich) in PBS for 15 min at RT in the dark. After two final PBS washes, slides were mounted using ProLong Glass Antifade Mountant (Thermo Fisher Scientific) and cured for at least 30 min before imaging on a Nikon C2 microscope.

#### Immunofluorescence combined to RNA-FISH

For *DDERMMAL*-FISH combined with immunofluorescence of PARP-1, the fluorescent hybridization protocol described previously was executed with the following modifications. After permeabilization and washing in wash buffer, cells were blocked for 30 min at RT in blocking buffer, as per the immunofluorescence protocol. Incubation with Anti-PARP-1 antibody (ab227244, 1:1000, Abcam) was then carried out in hybridization buffer together with *DDERMMAL*-FISH probes (125 nM, Biosearch Technologies) at 37°C overnight. Following the post-hybridization wash, cells were incubated with Alexa Fluor 647 secondary antibody (Thermo Fisher Scientific, 1:1000 in blocking buffer) at RT for 45 min in the dark. The remaining steps of the immunofluorescence protocol were then followed, and imaging was performed using a Zeiss Celldiscoverer 7 Airyscan microscope with a 50X water immersion objective.

#### Fluorescent detection of total RNA content

To stain for total RNA content, SytoRNA Select Green fluorescent dye (catalog number S32703, Thermo Fisher Scientific) was used following the manufacturer’s recommendations. Briefly, SKMEL28 cells were cultured on round glass coverslips (VWR) and subjected to either *DDERMMAL* knockdown (si*DDERMMAL*) or control conditions (siCtrl) for 72 h. Cells were then fixed in pre-chilled methanol for 10 min and washed three times for 5 min each with PBS. Next, cells were incubated with the SytoRNA Select fluorescent cell stain (500 nM in PBS) for 20 min at room temperature. Three additional 5-min PBS washes were performed before counterstaining with DAPI (1:1000 in PBS, Sigma-Aldrich). Finally, samples were mounted using ProLong Glass Antifade Mountant (Thermo Fisher Scientific) and cured for at least 30 min before imaging on a Nikon C2 microscope. Images were quantified using Fiji.

#### Comet assay

To assess DNA damage at the single-cell level, comet assays were performed using the OxiSelect Comet Assay Kit (Cat# STA-351, Cell Biolabs) according to the manufacturer’s instructions. SKMEL28 cells subjected to *DDERMMAL* knockdown (si*DDERMMAL*) or stable overexpression (*DDERMMAL*-OE), together with their respective controls (siCtrl and empty vector, EV), were treated with 250 nM doxorubicin for 16 h. Cells were then scraped, centrifuged at 700 × g for 2 min, and the resulting pellet washed once with ice-cold PBS before a second centrifugation, after which the supernatant was discarded. Pellets were resuspended at 1 × 10^5^ cells/mL in ice-cold PBS and mixed with molten agarose at a 1:10 (v/v) ratio. The suspension was immediately spread onto pre-warmed comet slides (37°C) to ensure full coverage of the wells. Slides were allowed to solidify at 4°C, then immersed for 1 h at 4°C in lysis buffer (14.6 g NaCl, 20 mL EDTA solution, 10 mL 10× lysis solution, 10 mL DMSO, DI H_2_O to 100 mL; pH 10 adjusted with 10 N NaOH). Slides were subsequently incubated for 30 min at 4°C in the dark with alkaline buffer (1.2 g NaOH, 0.2 mL EDTA solution, DI H_2_O to 100 mL) to relax and denature DNA. Electrophoresis was then carried out in pre-chilled alkaline electrophoresis buffer (300 mM NaOH, pH > 13, 1 mM EDTA in DI H_2_O) using a horizontal chamber, allowing fragmented DNA to migrate and form comet tails. Following electrophoresis, slides were washed three times in pre-chilled DI H_2_O, once in cold 70% ethanol, then dried completely at 37°C for 45 min. DNA was stained with Vista Green dye for 15 min and visualized by epifluorescence microscopy (Zeiss Discovery V8) and confocal microscopy (Nikon C2). DNA damage was quantified using Tail DNA % and Tail Moment measurements with the OpenComet plugin in Fiji.

#### RNA Affinity Purification (RAP)

50 μL of Pierce Streptavidin Magnetic Beads (Thermo Fisher Scientific) were coupled with 400 pmol of 24 biotinylated probes (Biosearch Technologies) targeting *DDERMMAL* (Table S1) and incubated overnight at 4°C with continuous rotation. As a negative control, pooled biotinylated probes (IDT) specific to the prostate-specific lncRNA *PCA3* (Table S1) were used. MM001 cells were lysed in 3 mL of pull-out buffer (20 mM Tris-HCl, pH 7.5, 200 mM NaCl, 2.5 mM MgCl_2_, 0.05% NP-40, 60 U/mL SUPERase·In RNase inhibitor, 1 mM DTT, and 1X Halt Protease and Phosphatase Inhibitor Cocktail) by rotating for 1 h at 4°C. The lysate was centrifuged at 14,000 × g for 10 min at 4°C to pellet membranes. For each RAP, 460 μg of RNA was incubated with the probe-bead mixture for 3 h and 30 min at 4°C with rotation.

Following incubation, the beads were washed six times with lysis buffer. One-third of the beads were subjected to RNA extraction using TRIzol, while the remaining two-thirds were eluted in Laemmli buffer by boiling at 95°C for 15 min with vigorous shaking. The Laemmli eluates were subsequently loaded onto SDS-PAGE gels for mass spectrometry analysis (see section below). The experiment was validated by qPCR and western blot analysis.

For RAP with UV crosslinking, cells were washed with PBS, and all liquid was removed by gently shaking the plates dry. Plates were then placed in a UVP crosslinker (Analytik Jena, France) without lids, and cells were irradiated with UV at 4 mJ/cm^2^ (265 nm). Cells were immediately scraped into ice-cold PBS, spun down at 300g for 5 min at 4°C, and then lysed in pull-out buffer before incubation with the probe-bead mixture as previously described. After incubation, the beads were washed six times, alternating between pull-out buffer and DEPC water for increased stringency. Beads were divided as before for RNA and protein elution. RNA was decrosslinked by incubation at 55°C for 15 min with mild shaking in 150 μL of decrosslinking buffer (100 mM Tris-HCl, pH 7.5, 50 mM NaCl, 10 mM EDTA, 0.5% SDS, 60 U/mL SUPERase·In RNase inhibitor, and 1 mg/mL Proteinase K).

#### Mass spectrometry analysis of RAP experiments

*DDERMMAL* and control (lncRNA *PCA3*) samples were analyzed in three independent biological replicates. Samples were separated by SDS-PAGE (NuPAGE 4–12% Bis-Tris Gel, Invitrogen) and stained with Coomassie Brilliant Blue (InstantBlue, Abcam). Five consecutive gel bands per lane were excised and subjected to in-gel tryptic digestion as previously described.^57^ Proteins were reduced with 10 mM DTT for 1 h at 56°C and alkylated with 55 mM iodoacetamide for 45 min at room temperature in the dark. Tryptic digestion (12.5 ng/μL) was performed overnight at 37°C. Peptides were extracted from gel pieces using 3% trifluoroacetic acid (TFA) and 30% acetonitrile (ACN), desalted on homemade STAGE-Tip microcolumns^58^, and eluted in 40 μL of buffer B (80% ACN, 0.1% formic acid). Eluates were dried in a SpeedVac concentrator (Eppendorf) and resuspended in 5 μL of 1% TFA prior to LC-MS/MS analysis. Peptide separation was performed on an EASY-nLC 1200 system (Thermo Fisher Scientific) coupled to a Q Exactive PLUS mass spectrometer (Thermo Fisher Scientific) via an EASY-Spray nano-electrospray ion source. The system was operated in single-column mode using a 25 cm EASY-Spray PepMap RSLC C18 column (Thermo Fisher Scientific) at 45°C. Solvent A was 0.1% FA in water, and solvent B was 0.1% FA in 80% ACN. Peptides were separated at 250 nL/min using a gradient of 5–20% B over 29 min, 20–30% B for 10 min, and 30–65% B for 5 min. The mass spectrometer was operated in data-dependent acquisition (DDA) mode. The 10 most intense precursor ions (charge ≥2) were isolated (target = 3 × 10^6^) and fragmented by HCD at a normalized collision energy of 28%. Full MS scans were acquired in the Orbitrap at 70,000 resolution (m/z 200) over a 375–1550 m/z range, with maximum injection times of 20 ms (MS) and 100 ms (MS/MS). Dynamic exclusion was set to 20 s. Raw data were processed with MaxQuant (v1.6.2.3) using the Homo sapiens UniProt database (UP000005640). Enzyme specificity was set to trypsin with up to two missed cleavages. Minimum peptide length was 7 amino acids. Mass tolerances were 4.5 ppm for precursor ions (after recalibration) and 20 ppm for fragment ions. Methionine oxidation and N-terminal acetylation were set as variable modifications. FDR was controlled at 1% at both peptide and protein levels. Label-free quantification was performed using the MaxLFQ algorithm^59^ with a minimum ratio count of 2, and identifications were transferred between runs using the match-between-runs feature (0.7 min window).^60^ MaxQuant output files (ProteinGroups.txt) were analyzed in Perseus.^61^ Entries annotated as “reverse”, “only identified by site”, or “contaminant” were removed. Proteins with at least three valid values in one group (healthy or tumor) were retained. Missing values were imputed from a normal distribution (downshift = 1.8, width = 0.3). Log_2_-transformed LFQ intensities and *p*-values were visualized as volcano plots comparing Target vs. Control and Target vs. Pre-IP samples **(**Table S3**).**

#### String database analysis of proteins enriched upon RAP experiments

Protein–protein interaction (PPI) analysis was performed using the STRING database^39^ (version 12.0; https://string-db.org). The list of selected genes was uploaded to the platform with Homo sapiens (taxon ID 9606) as the reference organism. The network was generated including both direct (physical) and indirect (functional) associations, with a minimum required interaction score set to 0.7. Only proteins from the input list were included in the visualization. Network statistics, including the number of nodes and edges, average node degree, clustering coefficient, and the PPI enrichment *p*-value, were obtained as generated by STRING suite. The resulting network was visualized using the built-in STRING layout. Functional enrichment analysis of the network nodes was also performed within STRING, assessing Gene Ontology (GO) Biological Process and Molecular Function. For each enriched term, the number of associated genes, enrichment strength, and false discovery rate (FDR) were reported.

#### Protein extraction and western blot

To extract proteins, cell pellets were lysed in RIPA buffer [150 mM NaCl, 50 mM Tris-HCl (pH 8), 1% Nonidet P40 (Thermo Fisher Scientific), 0.5% Sodium Deoxycholate (Sigma-Aldrich), 1 mM EDTA (Sigma-Aldrich)] supplemented with 1X Halt Protease and Phosphatase Inhibitor Cocktail (Life Technologies) by incubation on a rotating wheel at 4°C for 1 h. Samples were then centrifuged at 14,000 × g for 10 min at 4°C to pellet cell membranes, and supernatants were collected. Protein concentration was measured using the Bradford protein assay.

Western blot experiments were conducted using the following primary antibodies with the indicated concentrations: anti-PARP-1 (ab227244, 1:500, Abcam); anti-GPNMB (D-9, 1:500, Santa Cruz); anti-AGO2 (11A9 hybridoma supernatant, kind gift from Gunter Meister); β-Actin (13E5, 1:5000, Cell Signaling Technology). The following HRP-linked secondary antibodies were used: α-mouse IgG (NA931- 1 ML, 1:5000, Sigma-Aldrich) and α-rabbit IgG (NA934-1 ML; 1:5000, Sigma-Aldrich).

#### Nuclear/cytoplasmic fractionation

Melanoma cells were seeded into 15 cm plates and kept in standard culture media for 72 h. Nuclear and cytoplasmic fractionation was performed using the Nuclei EZ Prep Kit (Sigma-Aldrich) according to the manufacturer’s instructions. Briefly, cells were washed with ice-cold Dulbecco’s Phosphate-Buffered Saline (PBS) and then harvested in PBS using a cell scraper, retaining 10% of the volume as input. Cells were centrifuged at 300 × g for 5 min at 4°C and lysed with 1.5 mL Nuclei EZ buffer, supplemented with SUPERase·In RNAse inhibitor (60 U/mL, Thermo Fisher Scientific), 1X Halt Protease and Phosphatase Inhibitor Cocktail (Life Technologies), and 1 mM DTT (Sigma-Aldrich). The lysate was briefly vortexed and placed on ice for 5 min, then centrifuged at 500 × g for 5 min at 4°C. The supernatant, representing the cytoplasmic fraction, was transferred to a new tube. The pellet representing the nuclear fraction was washed with 1.5 mL of the same buffer, briefly vortexed, incubated on ice for 10 min, and centrifuged again at 500 × g for 5 min at 4°C. A final wash was performed without further ice incubation. Both cytoplasmic and nuclear fractions, along with the input, were divided into two tubes each for subsequent protein and RNA extraction. Nuclear and cytoplasmic ratios were calculated following normalization to total RNA content. RT–qPCR for *MALAT1*and *16S* were used to confirm the purity of the fractions.

#### RNA immunoprecipitation (RIP)

For RIP, MM001 cells were lysed in 2 mL immunoprecipitation buffer [20 mMTris-HCl pH 8.0, 200 mM NaCl, MgCl2 2.5 mM, Triton X-100 1%, 1 mM DTT (Sigma), 60 U/ml RNAseIn and 1× Halt Protease and Phosphatase Inhibitor Single-Use Cocktail (Life Technologies)], the cells were then incubated for 1h at 4°C on a rotating wheel and then spun down at 14000 × g for 15 min at 4°C. Supernatant was collected and protein content measured with Bradford assay (Bio-Rad). 10% of the total lysate was kept for RNA and protein inputs. A minimum of 1 mg of protein was used for the RIP. PARP-1 and GPNMB were immunoprecipitated using 4 μg of anti-PARP-1 antibody (ab227244, Abcam) or 4 μg of anti-GPNMB antibody (D-9, Santa Cruz) overnight at 4°C on a rotating wheel. 4 μg of normal rabbit IgG (12–370, LOT: 3493998, Millipore) was used as control. For PARP-1 immunoprecipitation, 80 μL of Protein G Dynabeads (Invitrogen) were then added to the lysate and left on a rotating wheel for 3h30 at 4°C. For GPNMB immunoprecipitation, 60ul of Protein A Dynabeads (Invitrogen) were added and incubated following the same conditions. Beads where then washed several times with immunoprecipitation buffer. RNA and protein extracts were eluted as described in the RAP section. The experiment was validated through qPCR and western blot. The relative expression of the genes of interest in the RIP was determined using the ΔΔCt method. First, the Ct value of the Input sample was subtracted from the Ct value of the RIP sample for each gene, resulting in the ΔCt for the RIP sample. This ΔCt was then compared to the ΔCt of the IgG control by subtracting the IgG ΔCt, yielding the ΔΔCt value. The fold enrichment was calculated using the following formula: fold enrichment = 2^−ΔΔCt^

#### *DDERMMAL* cloning and lentivirus transduction

To clone minimal *DDERMMAL* into a PGK lentiviral vector (Addgene), *DDERMMAL* cDNA was amplified by PCR using cDNA derived from whole-cell lysates of MM001 cells as a template and the following primers: forward, CTGGAATTCCATGGCTGAGGTGCAACAGGTCCC; reverse, TGGCTTCTTGCCATGTGCTC. PCR was carried out using GoTaq G2 (Promega) according to the manufacturer’s instructions. The PCR product was purified and subsequently re-amplified with primers containing 26-nt overlaps with the empty vector: forward, CAAGTTTGTACAAAAAAGCAGGCTTCCTGGAATTCCATGG; reverse, ACCACTTTGTACAAGAAAGCTGGGTCTGGCTTCTTGCCAT. The pLenti PGK Puro DEST (w529-2) vector (Addgene), previously engineered to contain *SAMMSON* (synthesized by GenScript, as described elsewhere^55^), was linearized to remove the *SAMMSON* insert. The linearized vector and the minimal *DDERMMAL* PCR product, with designed overlapping ends, were assembled using Gibson Assembly (New England Biolabs) following the manufacturer’s protocol.

The assembled vector was transformed into NEB 10-beta Competent E. coli (High Efficiency) (New England Biolabs) and single colonies were grown in LB media supplemented with Ampicillin (100 μg/mL). Plasmids were then purified and sent for Sanger DNA sequencing to confirm the insertion of minimal *DDERMMAL* in the vector and verify the accuracy of its sequence. *DDERMMAL* sequence for overexpression (5′ to 3′) is as follows:

CTGGAATTCCATGGCTGAGGTGCAACAGGTCCCACAGCCTCTCCAGCCTCTGTGGGAAGAAGGATCATCTGATGCCTATGTAATAGAGTTGCTGAAGGAGTGGAAGCAGCTTTGACATTATCAGTGGAACCCACCAGTGCAGACTGCTTCACCAAGGGCACCTGAGGCAGCAGATCTGAGGATTAGGGACGTAGGGAGCTGAAGCCTGGCTGGAGAGGTGGTGCGCACAAGAACCCCCACATTCCCCGACAGGAGCCTGAAAAAGCAAGACGCCATGTTGGGAGGACACGGAAGCAGTTCTAGGGAGAGGAGCACATGGCAAGAAGCCA.

Empty vector and *DDERMMAL* lentiviruses were produced in HEK293 cells using the CalPhos Mammalian Transfection Kit (Takara) according to the manufacturer’s instructions. Viral supernatants were collected 48 h post-transfection, clarified by centrifugation and filtration, then concentrated using Vivaspin 20 centrifugal concentrators (Sartorius) and used to infect SKMEL28 cells. To select for successfully transduced cells, puromycin (2 μg/mL) was added 48 h post-infection, and the medium was refreshed 72 h later. Cells were then maintained under puromycin selection at 400 ng/mL.

#### TCGA patient data

RNA expression data and associated clinical and molecular information from melanoma patients were obtained from The Cancer Genome Atlas (TCGA_SKCM) dataset via the UCSC Xena browser. Patients were stratified into “*DDERMMAL*-high” and “*DDERMMAL*-low” groups based on the median expression level of *DDERMMAL*. Gene Set Enrichment Analysis (GSEA, v4.3.3) was then performed to evaluate enrichment of melanoma transcriptional states as defined by Rambow et al., 2018.^8^ Additionally, correlation analyses were performed among *DDERMMAL*, *MITF*, and *SOX10*.

### Quantification and statistical analysis

Experimental data are reported as mean ± SEM from at least three biological triplicates unless otherwise stated in figure legends. All statistical details are indicated in the figure legends. *p* values are represented as follows: *p* > 0.05, not significant (ns); *p* < 0.05, ∗*p* < 0.01, ∗∗*p* < 0.001, ∗∗∗*p* < 0.0001, ∗∗∗∗. Statistical significance was determined using GraphPad Prism.

## Supplemental information

Document S1. Figures S1–S4 and Tables S1, S2

Table S3. List of proteins interacting with DDERMMAL detected by mass spectrometry, related to Figure 4
