# Supplementary material for: DDERMMAL, a melanocytic long non-coding RNA, confers DNA damage tolerance to melanoma cells
Source: iScience. 2026 Jun 5;29(6):116251. doi: 10.1016/j.isci.2026.116251 (PMC13264319; doi:10.1016/j.isci.2026.116251)
Supplement: Document S1. Figures S1–S4 and Tables S1, S2 [file mmc1.pdf]

**iScience, Volume *29***

## Supplemental information

# *DDERMMAL*, a melanocytic long non-coding RNA, confers DNA damage tolerance to melanoma cells

**Sara Adnane, Yvessa Verheyden, Alessandro Cuomo, Tiziana Bonaldi, and Eleonora Leucci**

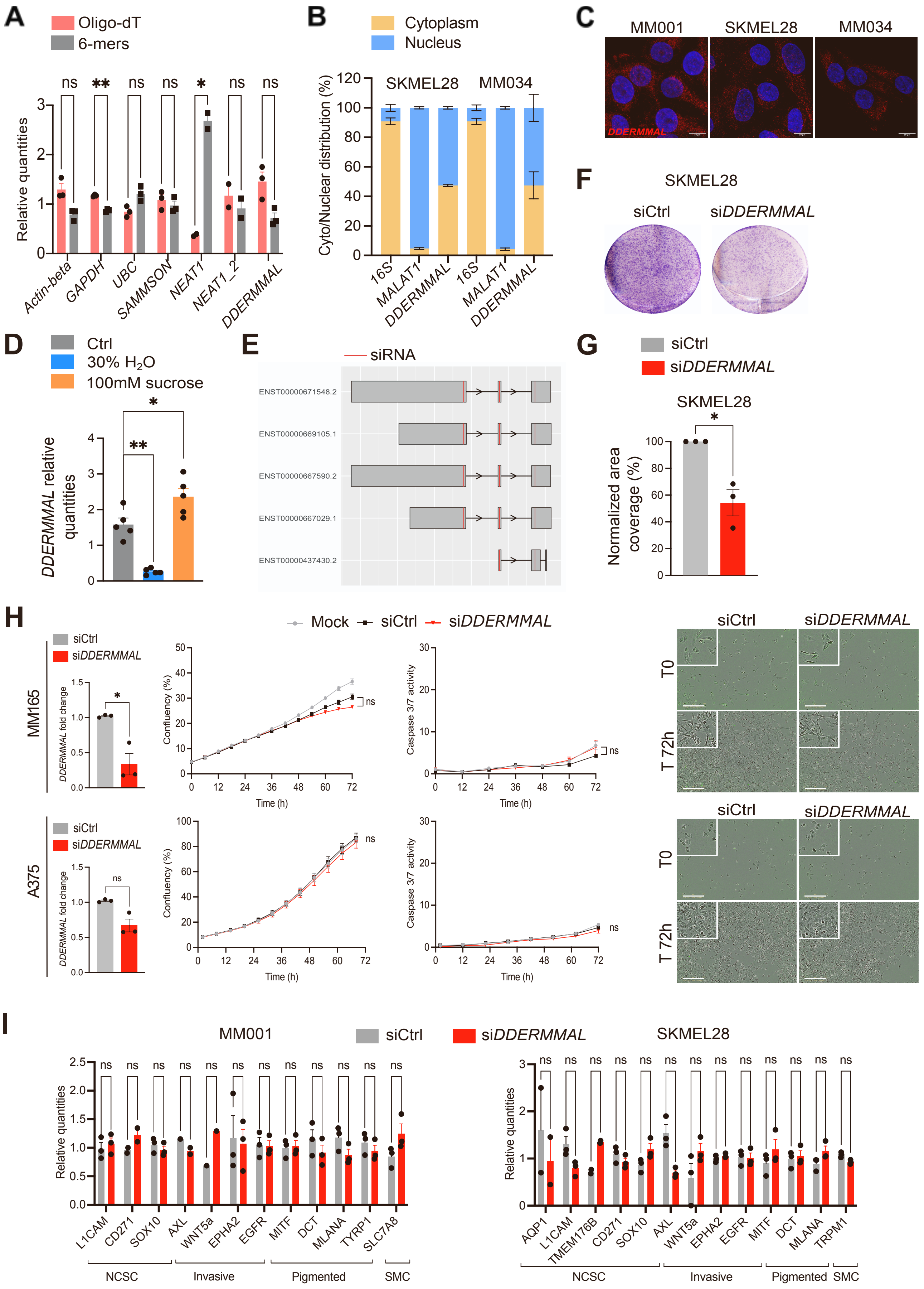


**Figure S1. Molecular and functional characterization of *DDERMMAL*. Related to Figure 1 and Figure 3.**

**A.** Relative quantification of *DDERMMAL* polyadenylation as assessed by comparing RT with oligo-dT or random hexamers priming prior of qPCR. Total *NEAT1* and *NEAT1*

long form have been used as negative and positive controls, respectively. Data are shown as mean ± SEM from independent biological replicates (n=3). Statistics were calculated by multiple paired t-test. **B.** Quantification of nuclear/cytoplasmic fractions of SKMEL28 and MM034 as assessed by RT-qPCR. *MALAT1* and *16S* were used as nuclear and cytoplasmic control respectively. Data are shown as mean ± SEM from independent biological replicates (n=3). **C.** FISH for *DDERMMAL* (red) in 3 melanocytic cell lines, as indicated in the top of the figure, upon normal conditions. Nuclei were counterstained with DAPI (blue). **D.** RT-qPCR showing relative *DDERMMAL* expression upon hypo- and hyper-osmotic stress in SKMEL28. Data are shown as mean ± SEM from independent biological replicates (n=5). Statistics were by multiple paired t-test. **E.** Schematic representation of AL159166.1 annotated transcript isoforms. In red the binding position of the siRNAs in the pool used for its silencing. **F.** Crystal violet viability assay of SKMEL28 upon *DDERMMAL* knockdown. **G.** Quantification of the viability assay in F expressed as percentage of area covered by the crystal violet staining. Data are shown as mean ± SEM from independent biological replicates (n=3). Statistics were calculated by paired t-test. **H.** RT-qPCR showing *DDERMMAL* knockdown efficiency in MM165 (top left) and A375 (bottom left). Statistical significance was calculated by paired t-test. Cell confluency and caspase 3/7 activation (middle panels) measured by live-cell imaging in MM165 (top middle) and A375 (bottom middle) cells upon *DDERMMAL* knockdown. Brightfield images (right) are representative of cells immediately after transfection (T0) and 72 h post-transfection (T72h). Scale bar= 300 µm. Data are shown as mean ± SEM from independent biological replicates (n=3). Statistical significance was assessed by paired t-test for RT–qPCR analyses and by two-way ANOVA with mixed-effects analysis for live-cell imaging assays. **I.** RT-qPCR for melanocytic and drug-tolerant markers in MM001 and SKMEL28 upon *DDERMMAL* knockdown. Data are shown as mean ± SEM from independent biological replicates (n=3). Statistical significance was calculated by ANOVA.

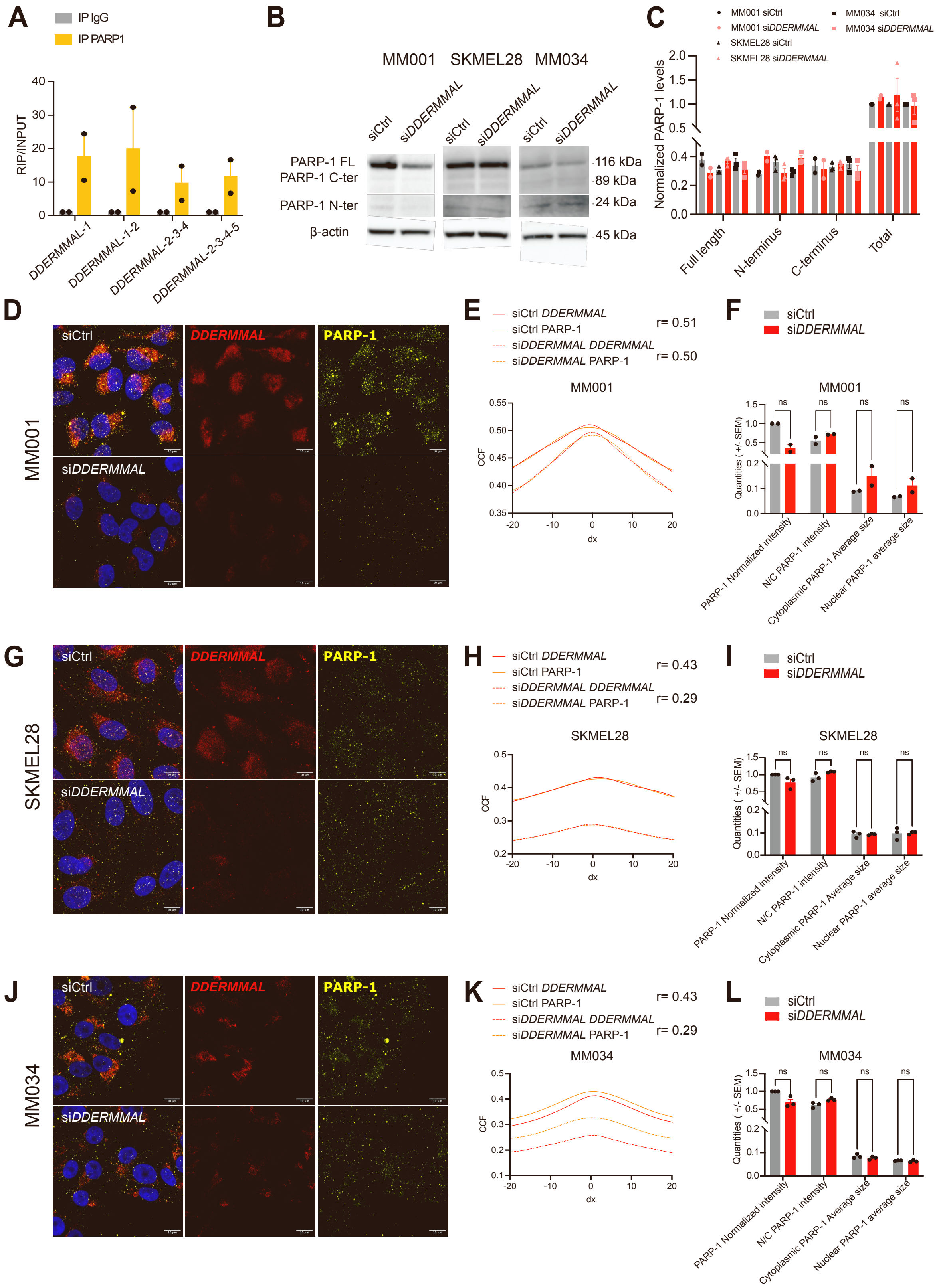


**Figure S2. *DDERMMAL* does not affect PARP-1 cleavage or subcellular localization. Related to Figure 4.**

**A.** *DDERMMAL* isoforms' enrichment over input in control (RIP IgG) and PARP-1-specific RNA Immune Precipitation (RIP PARP-1) as calculated by RT-qPCR in MM001. Data are shown as mean ± SEM from independent biological replicates (n=2).

**B.** Representative images of PARP-1 cleavage estimation as assessed by western blot performed with MM001, SKMEL28 and MM034 lysates derived from cells exposed to *DDERMMAL* knockdown. **C.** Quantification of the gels in B. Independent biological replicates were analysed for each cell line (MM001, n=2; SKMEL28, n=3; MM034, n=3). Statistics were calculated by multiple paired t-tests. **D. G. J.** Immune fluorescent staining and confocal imaging of PARP-1 (yellow) and *DDERMMAL* (red). Nuclei were counterstained with DAPI (blue). The name of the cell line tested is indicated on the left side of each panel. Scale bar= 10 μm. **E.H. K.** Colocalization between *DDERMMAL* and PARP-1 signal as measured by Cross-Correlation Function (CCF) using Jacop. R indicates the correlation coefficient. **F.I.L.** Quantification of PARP-1 staining from pictures in D, G and J. Data are shown as mean ± SEM from independent biological (MM001, n=2; SKMEL28, n=3; MM034, n=3). Statistics were calculated by multiple paired t-tests.

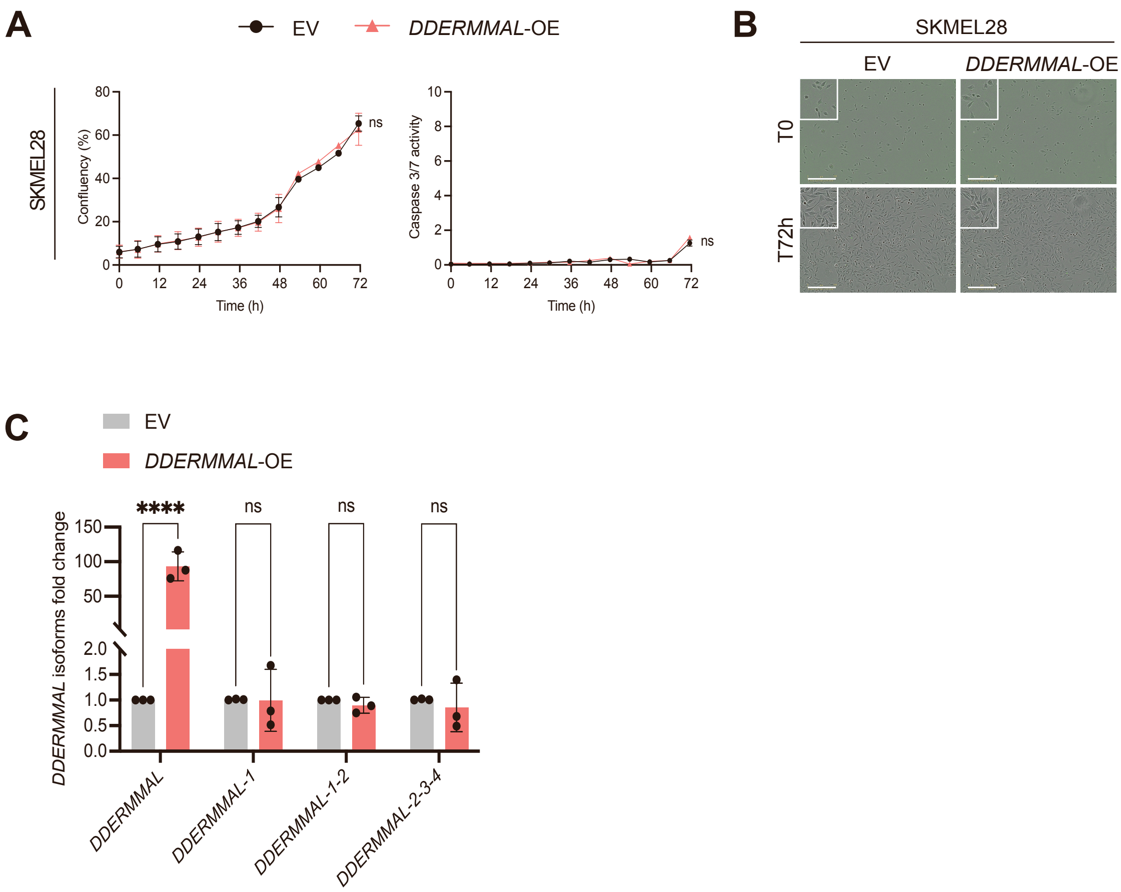


**Figure S3. Overexpression of *DDERMMAL* minimal isoform does not affect melanoma cell viability. Related to Figure 5.**

**A.** Cell confluency (left) and caspase 3/7 activation (right) measured by live-cell imaging in SKMEL28 cells stably overexpressing *DDERMMAL* compared with empty vector (EV) controls. Data are shown as mean ± SEM from independent biological replicates (n=3). Statistical significance was assessed by two-way ANOVA with mixed-effects analysis. **B.** Representative brightfield images of SKMEL28 cells at the start of the experiment (T0) and after 72 h of growth on the IncuCyte (T72h). Scale bar= 300 µm. **C.** RT-qPCR showing *DDERMMAL* overexpression efficiency in SKMEL28, expressed as fold change relative to empty vector control. Expression of all *DDERMMAL* isoforms was also measured to assess whether minimal isoform overexpression affects their levels. Data are shown as mean ± SEM from independent biological replicates (n=3). Statistical significance was calculated using two-way ANOVA.

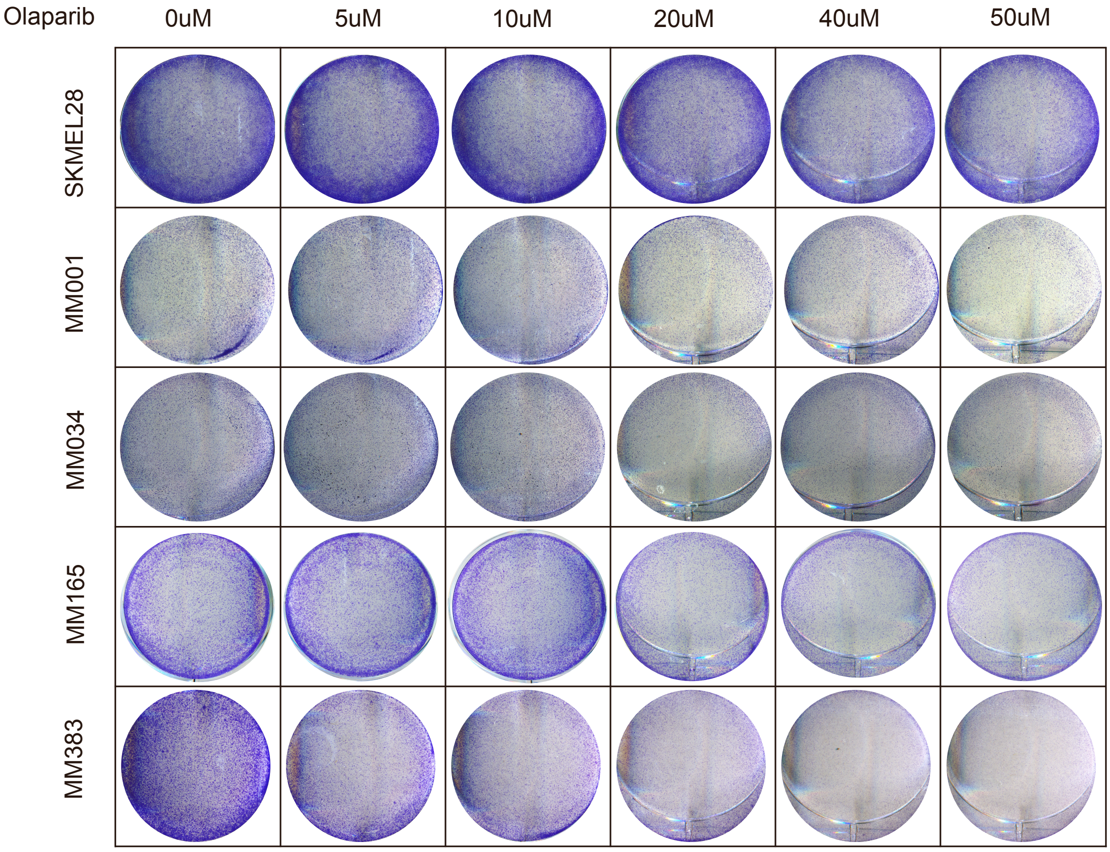


**Figure S4. Crystal violet viability assay of different melanoma cell lines upon exposure to increasing concentration of Olaparib for 24 h. Related to Figure 5.** Representative images from 3 independent biological replicates are shown.

| *DDERMMAL*-FISH probe sequence (5’ to 3’) | *DDERMMAL*-RAP probe sequence (5’ to 3’) | *PCA3*-RAP probe sequence (5’ to 3’) |
|---|---|---|
| TGAGAGGGGCTGCATTATTC-[TAMRA-C9] | TGAGAGGGGCTGCATTATTC-[biotin] | [biotin]-GCACTTGCTATTTCTTCTGT |
| TGGAATTCCAGACACTCCAT-[TAMRA-C9] | TGGAATTCCAGACACTCCAT-[biotin] | [biotin]-CTCTGTTTTTCTGATGCCAG |
| AAACCTTCAGTCACCTGTTG-[TAMRA-C9] | AAACCTTCAGTCACCTGTTG-[biotin] | [biotin]-GCTGCAGCCACACAAATCTC |
| TCTGCTTCTGACTGTGAGTG-[TAMRA-C9] | TCTGCTTCTGACTGTGAGTG-[biotin] | [biotin]-ATGCAGATCTTCCTGGTCTC |
| TCACATCTGGCCATCTCATC-[TAMRA-C9] | TCACATCTGGCCATCTCATC-[biotin] | |
| CAATCACAGGGTATCTGCAA-[TAMRA-C9] | CAATCACAGGGTATCTGCAA-[biotin] | |
| ATTGCATCTGGTTTACTTGC-[TAMRA-C9] | ATTGCATCTGGTTTACTTGC-[biotin] | |
| AGGACTTTCATGATGGGGAA-[TAMRA-C9] | AGGACTTTCATGATGGGGAA-[biotin] | |
| TGCCAACTCTGCATCATAAG-[TAMRA-C9] | TGCCAACTCTGCATCATAAG-[biotin] | |
| GAGATCCCAACAAGGGTCAG-[TAMRA-C9] | GAGATCCCAACAAGGGTCAG-[biotin] | |
| TAGACGTTCCAAGGCTCACA-[TAMRA-C9] | TAGACGTTCCAAGGCTCACA-[biotin] | |
| TGCAGCTCTAAATTTGGCTG-[TAMRA-C9] | TGCAGCTCTAAATTTGGCTG-[biotin] | |
| CCATGAATTAGCTTTGCTCA-[TAMRA-C9] | CCATGAATTAGCTTTGCTCA-[biotin] | |
| CAGGACTCAGTGTGAACTGA-[TAMRA-C9] | CAGGACTCAGTGTGAACTGA-[biotin] | |
| TCAGTCTCTTCCTACAGGTG-[TAMRA-C9] | TCAGTCTCTTCCTACAGGTG-[biotin] | |
| AGGGCCTTTTAATTATTGGG-[TAMRA-C9] | AGGGCCTTTTAATTATTGGG-[biotin] | |
| CCCTTTCATCCAGATTTAAG-[TAMRA-C9] | CCCTTTCATCCAGATTTAAG-[biotin] | |
| TGCACTTTGTAGCACAGACA-[TAMRA-C9] | TGCACTTTGTAGCACAGACA-[biotin] | |
| AAGAGACTGCGTATCAACGC-[TAMRA-C9] | AAGAGACTGCGTATCAACGC-[biotin] | |
| GTGGGTTAGTTAACAGGCAG-[TAMRA-C9] | GTGGGTTAGTTAACAGGCAG-[biotin] | |
| TTTATAAGTCCTTGCTCCTG-[TAMRA-C9] | TTTATAAGTCCTTGCTCCTG-[biotin] | |
| TGCACTTAACCAGCTCATAA-[TAMRA-C9] | TGCACTTAACCAGCTCATAA-[biotin] | |
| AATACTATGCCGAGATTGCC-[TAMRA-C9] | AATACTATGCCGAGATTGCC-[biotin] | |
| GCAGGGGAGGGAAAACGGAA-[TAMRA-C9] | GCAGGGGAGGGAAAACGGAA-[biotin] | |

**Table S1. Sequences of FISH and RAP probes. Related to Figure 4 and STAR Methods.**

| Target | Forward primer (5’ to 3’) | Reverse primer (5’ to 3’) |
|---|---|---|
| *16S* | CTCGATGTTGGATCAGGACA | CCTGGATTACTCCGGTCTGA |
| *Actin-Beta* | AGCGAGCATCCCCCAAAGTT | GGGCACGAAGGCTCATCATT |
| *AQP1* | TGGACACCTCCTGGCTATTGAC | AGACCCCTTCTATTTGGGCTT |
| *AXL* | CAATGGGGACTACTACCGCC | GAAGGACCACACATCGCTCT |
| *CD271* | TCATCCCTGTCTATTGCTCCA | TGTTCTGCTTGCAGCTGTTC |
| *DCT* | TATTAGGACCAGGACGCCCC | TGGTACCGGTGCCAGGTAAC |
| *DDERMMAL* | TTAGGGACGTAGGGAGCTGA | GGCGTCTTGCTTTTTCAGGC |
| *DDERMMAL-1* | GTTGGTGGCACTTTTTGGCT | CACTGTGGAGTGCGGTTTTC |
| *DDERMMAL-1-2* | TCTCCTCATTTCATGACAGGCT | CCTTTTCTGGGGGAGGGGATT |
| *DDERMMAL-2-3-4* | AAGGTAGGGAAGGGTCTCTCC | TGAGAGGGGCTGCATTATTCC |
| *EGFR* | CCAAACTGCACCTACGGATG | ACGGGATCTTAGGCCCATTC |
| *EPHA2α* | CTCACACACCCGTATGGCAA | GAAGTTGGTGCCGTAGTCCA |
| *GAPDH* | GTCAGCCGCATCTTCTTTTG | GCGCCCAATACGACCAAATC |
| *HPRT* | AGCCAGACTTTGTTGGAT TTG | TTTACTGGCGATGTCAATAAG |
| *L1CAM* | CTGCCTGCTTATCCAGATCC | CCTCACACTTGAGGCTGATG |
| *MALAT1* | GCTCTGTGGTGTGGGATTGA | GTGGCAAAATGGCGGACTTT |
| *MITF* | GAA GTC CTT AAG GTG CAG AC | GCT TGC TGT ATG TGG TAC TTG |
| *MLANA* | GCT CAT CGG CTG TTG GTA TTG | CAC TTT GCT GTC CCG ATG ATC |
| *NEAT1* | GTG GCT GTT GGA GTC GGT AT | TAACAAACCACGGTCCATGA |
| *NEAT1-2* | GTCTTTCCATCCACTCACGTCTATTT | GTACTCTGTGATGGGGTAGTCAGTCAG |
| *PCA3* | GAGGCCACACATCTGCTGAA | ATGTCCTTCCCTCACAAGCG |
| *ROSALIND* | GCAGTGTAGAAAGCACCCCA | AAAACCCCACGGGAAACTGT |
| *SAMMSON* | CCTCTAGATGTGTAAGGGTAGT | TTG AGT TGC ATA GTT GAG GAA |
| *SLC7A8* | TTCTATGGGGTCACGGTTGC | GGGTACACGACCACACACAT |
| *SOX10* | TAC CCG CAC CTG CAC AAC | TTC AGC AGC CTC CAG AGC |
| *TBP* | CGGCTGTTTAACTTCGCTTC | CACACGCCAAGAAACAGTGA |
| *TMEM176B* | CCCTACCACTGGGTACAGATGGA | CTT CAA GAC ACA GAC AGC CAG GA |
| *TRPM1* | CAC CCA GAG CTA CCC AAC AGA | CGG ATA TAC ATG GCT TTA TTG GAA |
| *TYRP1* | CAT GCA GGA AAT GTT GCA AGA G | AGT TTG GGC TTA TTA GAG TGG AAT |
| *UBC* | ATTTGGGTCGCGGTTCTT | TGCCTTGACATTCTCGATGGT |
| *WNT5A* | GGT GGT CGC TAG GTA TGA ATA ACC | TCC ACC TTC GAT GTC GGA A |

**Table S2. qPCR primer sequences. Related to STAR Methods.**
